# Comparative Analysis of Chloroplast Genome and New Insights Into Phylogenetic Relationships of *Polygonatum* and Tribe Polygonateae

**DOI:** 10.3389/fpls.2022.882189

**Published:** 2022-06-24

**Authors:** Jing Wang, Jun Qian, Yuan Jiang, Xiaochen Chen, Baojiang Zheng, Shilin Chen, Fajian Yang, Zhichao Xu, Baozhong Duan

**Affiliations:** ^1^College of Pharmaceutical Science, Dali University, Dali, China; ^2^Heilongjiang Key Laboratory of Plant Bioactive Substance Biosynthesis and Utilization, College of Life Science, Northeast Forestry University, Harbin, China; ^3^Key Laboratory of Beijing for Identification and Safety Evaluation of Chinese Medicine, Institute of Chinese Materia Medica, China Academy of Chinese Medical Sciences, Beijing, China; ^4^Baoshan College of Traditional Chinese Medicine, Baoshan, China

**Keywords:** *Polygonatum*, chloroplast genome, phylogenetics, divergence time, Trib. Polygonateae

## Abstract

Members of *Polygonatum* are perennial herbs that have been widely used in traditional Chinese medicine to invigorate Qi, moisten the lung, and benefit the kidney and spleen among patients. However, the phylogenetic relationships and intrageneric taxonomy within *Polygonatum* have long been controversial because of the complexity of their morphological variations and lack of high-resolution molecular markers. The chloroplast (cp) genome is an optimal model for deciphering phylogenetic relationships in related families. In the present study, the complete cp genome of 26 species of Trib. Polygonateae were *de novo* assembled and characterized; all species exhibited a conserved quadripartite structure, that is, two inverted repeats (IR) containing most of the ribosomal RNA genes, and two unique regions, large single sequence (LSC) and small single sequence (SSC). A total of 8 highly variable regions (*rps16-trnQ-UUG, trnS-GCU-trnG-UCC*, *rpl32-trnL-UAG*, *matK-rps16*, *petA-psbJ, trnT-UGU-trnL-UAA*, *accD-psaI*, and *trnC-GCA-petN*) that might be useful as potential molecular markers for identifying *Polygonatum* species were identified. The molecular clock analysis results showed that the divergence time of *Polygonatum* might occur at ∼14.71 Ma, and the verticillate leaf might be the ancestral state of this genus. Moreover, phylogenetic analysis based on 88 cp genomes strongly supported the monophyly of *Polygonatum*. The phylogenetic analysis also suggested that *Heteropolygonatum* may be the sister group of the *Polygonatum*, but the *Disporopsis, Maianthemum*, and *Disporum* may have diverged earlier. This study provides valuable information for further species identification, evolution, and phylogenetic research of *Polygonatum*.

## Introduction

*Polygonatum* Mill (1754) is an essential medicinal and edible species widely distributed in warm–temperate zones of the Northern Hemisphere and Northeastern Asia (Floden, 2015). There are approximately 70 species recognized worldwide (Floden and Schilling, 2018), with 39 present in China, 20 of which are endemic to the region (Chen and Tamura, 2000). The underground rhizomes of *Polygonatum* have crucial medicinal value in moistening lungs, relieving thirst, replenishing the spleen, and increasing immunity (Jiao et al., 2018a). Among them, four species [*Polygonatum odoratum* (Mill.) Druce, *Polygonatum sibiricum* Red., *Polygonatum cyrtonema* Hua, and *Polygonatum kingianum* Coll. et Hemsl] were listed in the Chinese Pharmacopoeia (Chinese Pharmacopoeia Commission, 2020). Modern studies have demonstrated that some *Polygonatum* species were rich in nutrients and functional components and were regarded as a new enormous potential miscellaneous grain (Si and Zhu, 2021). The previous survey has revealed that *Polygonati rhizoma* is often contaminated with several common adulterants in herbal markets, such as *Polygonatum cirrhifolium*, *Polygonatum humile*, *Polygonatum stenophyllum*, *Polygonatum filipes*, and *Polygonatum verticillatum* (Yang et al., 2015; Jiao et al., 2018b; Wang Y. et al., 2019; Wang Z. W. et al., 2019). Because the morphology of these species is similar, changeable, and indistinguishable, it seriously affects the safety and effectiveness of clinical drug use (Ali et al., 2021).

In addition, the phylogenetic position of *Polygonatum* has been controversial for many years. Some previous taxonomy places the genus within Convallariaceae, Ruscaceae, and Asparagaceae based on morphological and molecular phylogenies (Tang, 1978; Rudall et al., 2000; Kim et al., 2012). Concerning the intraspecific relationship of *Polygonatum*, according to the leaf order, Baker and Esq (1875) divided it into three groups: section (sect.) *Alternifolia* (=sect. *Polygonatum*), sect. *Verticillata*, and sect. *Oppositifolia*, but Tang (1978) considered that the classification mentioned above might be inappropriate for identification. He divided the genus into eight series (ser.): including ser. *Alternifolia*, ser. *Altelobata*, ser. *Bracteata*, ser. *Punctata*, ser. *Kingiana*, ser. *Hookeriana*, ser. *Verticillata*, and ser. *Oppositifolia*. Tamura (1993) considered that *Polygonatum* could be divided into sect. *Polygonatum* and sect. *Verticillata* according to stamen morphology, chromosome number, karyotype, and filament. Based on the *rpl16* gene and *trnK* gene, Wu et al. (2000) found that the opposite leaves and verticillate leaves of *Polygonatum* were polyphyletic. More recent molecular phylogenies based on one to several genes have suggested that the *Polygonatum* could be divided into three groups (Meng et al., 2014; Floden and Schilling, 2018), recommended that the *petA-psbJ* plastid gene region is combined with the nuclear ribosomal *ITS* for *Polygonatum* identification. However, the phylogenetic position of some species [i.e., *P. franchetii* Hua, *P. alternicirrhosum* Hand.-Mzt., *P. verticillatum* (L.) All., *P. punctatum* Royle ex Kunth] is in dispute (Meng et al., 2014; Floden, 2017; Floden and Schilling, 2018; Jiao, 2018; Zhao et al., 2019; Xia et al., 2022). Moreover, the current taxonomy of Tribe (Trib.) Polygonateae has long been debated, which has been divided into three to eight genera. For example, the genus was divided into eight genera: *Polygonatum, Disporopsis, Smilacina, Maianthemum, Disporum, Clintonia*, and *Streptopus* (Tang, 1978). Whereas Tamura et al. (1997) proposed three genera: *Polygonatum, Disporopsis*, and *Heteropolygonatum*. Thus, the boundaries and relationships of Trib. Polygonateae remain problematic.

Despite these potential issues, using the chloroplast (cp) genome for phylogenetic estimates generally shows promise for resolving deep relationships among the plant lineages (Nie et al., 2020). Compared with the traditional DNA fragments, the cp genome was relatively conserved and slightly varied (Liang et al., 2020). The method has recently been applied to many research fields, such as taxonomic revision, systematic evolution, and species identification (Chen et al., 2019; Henriquez et al., 2020). Floden and Schilling (2018) and Xia et al. (2022) used cp genome data to reconstruct the phylogeny of *Polygonatum*, and the results supported the three groups and their sister relationship with *Heteropolygonatum*. Although these studies resolved the phylogenetic relationships of some species of *Polygonatum*, the phylogenetic relationships among the genera of Trib. Polygonateae and some species of *Polygonatum* were still unclear. In addition, the reliability of some analyses still needs to be further clarified due to the limited number of samples in the previous study. Given this, it is necessary to provide further support for the intra-generic relationships, divergence times, and genomic characteristics of Trib. *Polygonateae* based on a larger sample size. In the present study, we *de novo* assembled and annotated the cp genome of 26 species, including 23 species of Asparagaceae (18 species of *Polygonatum*, four species of *Disporopsis*, and one species of *Maianthemum*), and three species of *Disporum* (Colchicaceae). Besides, comparative analysis and phylogenetic evolution of the cp genome were conducted. The present results provide a basis for species identification, phylogenetic studies, resource development, and utilization of *Polygonatum* medicinal plants.

## Materials and Methods

### Plant Material and DNA Sequencing

The fresh and healthy leaves of *Polygonatum, Disporopsis, Maianthemum*, and *Disporum* were collected in the field or Germplasm Resource Garden (China), and then the leaf tissue was frozen fresh at –20°C. Numbers after taxa names refer to the locality, and sample information is shown in Supplementary Figure 1 and Supplementary Table 1. The specimens were identified following the taxonomic key and external morphology diagnosis proposed by related literature (Tang, 1978). The voucher specimens have been deposited at the herbarium of Dali University. Total genomic DNA was extracted from tissue samples using the Plant Genomic DNA kit (Tiangen, Beijing, China). The extracted DNA was quantified on a Nanodrop 2000 spectrophotometer (Nanodrop Technologies, Thermo Scientific, United States), and all PCR products were tested for the presence of amplified products on agarose gels. The library of each sample was prepared using 30 μl of high-quality (>100 ng/) genomic DNA. All libraries were sequenced on the Illumina NovaSeq system (Illumina, San Diego, CA, United States).

### Genome Assembly and Annotation

The pair-end reads were trimmed for adapter and low-quality reads (Phred score < 30) using NGS QC Toolkit v.2.3.3 software. The cp genomes of *P. sibiricum* (NC029485), *Disporopsis fuscopicta* (MW248136), *Maianthemum bicolor* (NC035970), and *Disporum cantoniense* (MW759302) were downloaded from the National Center of Biotechnology Information (NCBI). The genome above was then used as the reference sequence. The cp genome was assembled using GetOrganelle v.1.6.4, exploiting Bowtie2 v.2.4.4, SPAdes v.3.13.0, and Blast v.2.5.0 as dependencies (get_organelle_from_reads.py -1 R1.fq -2 R2.fq -o cp_output -R 15 -k 21,45,65,85,105 -F embplant) (Jin et al., 2020). All clean reads were mapped to the database, and then the mapping data were extracted based on similarity and coverage. Subsequently, the assembled contigs were visualized and removed redundant sequence by Bandage v.0.8 to generate the complete circular cp genome (Wick et al., 2015). Finally, the reads were remapped to assembled cp genome by Bowtie2, and Jellyfish v.2.2.3 was then used to determine the reverse repeat region boundary. After assembly, circular cp genomes were annotated using online tools CpGAVAS2 and GeSeq based on the reference cp genome (Michael et al., 2017; Shi et al., 2019). The Apollo was used to correct the start codons, stop codons, and intron/exon boundaries (Lewis et al., 2002). Annotated cp genome sequences were submitted to the GenBank database of the NCBI to obtain specific accession numbers (Table 1). Fully annotated cp genome circle diagrams were drawn by OrganellarGenomeDRAW (OGDRAW) online (Lohse et al., 2007).

**TABLE 1:** Summary of cp genome features.

Species	Total length (bp)	GC content (%)	AT content (%)	LSC length (bp)	SSC length (bp)	IR length (bp)	Gene number	Protein-coding gene number	rRNA gene number	tRNA gene number	GenBank accession
*P. kingianum*	155,802	37.7	62.3	84,625	18,525	26,326	133	85	8	38	MZ029091
*P. cirrhifolium*	156,021	37.6	62.4	84,618	18,573	26,415	133	87	8	38	MZ029092
*P. sibiricum 1*	155,512	37.7	62.3	84,533	18,417	26,281	134	88	8	38	MZ029093
*P. cyrtonema*	155,512	37.7	62.3	84,462	18,292	26,379	132	86	8	38	MZ029094
*P. alternicirrhosum*	155,806	37.7	62.3	84,588	18,524	26,347	131	85	8	38	OL405009
*P. filipes*	155,472	37.7	62.3	84,422	18,292	26,379	133	87	8	38	OL405010
*P. franchetii*	155,228	37.7	62.3	84,164	18,418	26,323	133	87	8	38	OL405011
*P. hookeri*	155,953	37.6	62.4	84,573	18,550	26,415	133	87	8	38	OL405012
*P. humile*	155,185	37.7	62.3	84,102	18,455	26,314	133	87	8	38	OL405013
*P. hunanense*	155,456	37.7	62.3	84,286	18,426	26,372	133	88	8	38	OL405014
*P. involucratum*	155,372	37.7	62.3	84,282	18,450	26,320	133	87	8	38	OL405015
*P. odoratum*	154,576	37.8	62.2	83,493	18,459	26,312	133	87	8	38	OL405016
*P. prattii*	155,887	37.6	62.4	84,503	18,554	26,415	133	87	8	38	OL405017
*P. mengtzense*	155,590	37.7	62.3	84,539	18,427	26,312	131	85	8	38	OL587680
*P. stewartianum*	155,847	37.7	62.3	84,629	18,524	26,347	131	85	8	38	OL405018
*P. uncinatum*	155,681	37.7	62.3	84,596	18,529	26,278	133	87	8	38	OL405019
*P. sibiricum 2*	155,514	37.7	62.3	84,536	18,416	26,281	133	87	8	38	OL405024
*P. zanlanscianense*	155,827	37.6	62.4	84,463	18,534	26,415	133	87	8	38	OL405020
*P. stenophyllum*	155,961	37.7	62.3	84,609	18,561	26,395	132	86	8	38	OL405025
*Disporopsis aspersa*	156,110	37.7	62.3	85,055	18,525	26,265	131	85	8	38	OL405021
*Disporopsis longifolia*	156,008	37.7	62.3	85,048	18,480	26,280	131	85	8	38	OL405022
*Disporopsis fuscopicta*	155,934	37.7	62.3	84,923	18,527	26,242	131	85	8	38	OL405023
*Disporopsis pernyi*	156,072	37.7	62.3	85,017	18,525	26,265	131	85	8	38	OL587681
*Disporum megalanthum*	156,583	37.6	62.4	84,973	18,020	26,795	127	81	8	38	OL405026
*Disporum uniflorum*	156,588	37.6	62.4	84,977	18,023	26,794	127	81	8	38	OL405027
*Disporum cantoniense*	156,562	37.6	62.4	84,926	18,002	26,815	127	81	8	38	OL587682
*M. fuscum*	156,711	37.6	62.4	85,218	18,447	26,523	131	85	8	38	OL405028

### Genome Structure and Comparisons Analysis

The GC content was analyzed using Geneious v.9.0. Four types of the dispersed repeat sequence, including forward (F), complementary (C), palindromic (P), and reverse (R), were detected using the REPuter program^1^ (Stefan et al., 2001). Tandem repeats were detected using Phobos v.3.3.12^2^ (Mayer et al., 2010) with default parameter values. The cp genome of *P. sibiricum* (MZ029093) was selected as a reference for coordinate positions, and indels and SNPs were counted within the non-overlapping 150 bp window for 18 *Polygonatum* plastomes (Supplementary Table 7a; Liu et al., 2020). The region IRb was removed for the analyses of repeats to avoid over-representing the repeats following Abdullah et al. (2019). Spearman’s Rho correlations were calculated based on substitutions, Indels, and oligonucleotide repeats using Minitab v. 18 (Akoglu, 2018).

The criteria for repeat determination include a minimum repeat size of 20 bp with the similarity between repeat pairs of 90% by putting edit value 3. Furthermore, the simple sequence repeats (SSRs) were analyzed using MISA software^3^ with the parameters of “10” for mono-, “5” for di-, “4” for tri-, and “3” for tetra- and penta- nucleotide motifs (Beier et al., 2017). The cp genomes were compared with mVISTA under the Shuffle-LAGAN mode. The cp genome junctions were visualized and compared using IRscope^4^ online (Amiryousefi et al., 2018). The cp genomes were aligned using the MAFFT (Katoh and Standley, 2013). Additionally, the nucleotide variability across the cp genome sequences was analyzed using DnaSP v.6.12.03, with a window length of 600 sites and a step size of 200 sites.

### Phylogenetic Analyses and Ancestral Character State Reconstruction

Phylogenetic reconstruction included 27 *de novo* assembled sequences (Table 1), and 61 cp genomes downloaded from NCBI (Supplementary Table 2). At the same time, two species, *Dioscorea esculenta* (NC052854) and *Dioscorea schimperian* (NC039855), were used as outgroups. A total of 88 sequences were aligned using MAFFT with default parameters and trimmed using trimAl v.1.4 with option automated. Neighbor-Joining (NJ) analyses were performed using the MEGA X, applied with 1,000 bootstrap replicates at each branch node (Sudhir et al., 2018). The alignment was also evaluated using bootstrap analysis on 1,000 in a maximum likelihood (ML) by IQ-tree (Nguyen et al., 2015), with parameters: iqtree -s input -m MFP -b 1000 -nt AUTO -o NC052854, NC039855, best-fit nucleotide substitution model.

The leaf arrangement was selected to analyze the phyllotaxy evolution of *Polygonatum*. The phyllotaxy information was obtained from taxonomic literature and the Flora of China (Chen and Tamura, 2000; Jiao, 2018). The states of phyllotaxy were coded: alternate (A), verticillate (B), opposite (C), and the crowd (D). In the case of some species with more than two-character states, we coded the character state based on their dominant status. For example, the leaf arrangement for *Polygonatum prattii* was coded as alternate because it usually has alternate leaves, although there is an opposite leaf arrangement or three-verticillate leaves occasionally. For *Polygonatum hunanense*, its leaves were mainly verticillate, sometimes with a few alternate or opposite leaves, thus coded as verticillate in the analyses. Statistical Dispersal-Vicariance Analysis (S-DIVA) and Multistate Reconstruction BayesTraits (MRBT) method were conducted in RASP software to infer ancestral character states (Yu et al., 2015).

### Divergence Time Estimation

The divergence times of *Polygonatum* were calculated using the Markov chain Monte Carlo (MCMC) tree program of PAML (Puttick, 2019). IQ-tree was used to estimate the best tree topology of the data set. According to the previous study (Chen et al., 2013; Eguchi and Tamura, 2016; Wang et al., 2016; Xia et al., 2022), we used four calibration points to restrict each node: (F1) 115.9–137.4 Ma for the root node, (F2) 58.3–76.6 Ma for Asparagaceae stem age, (F3) 56.4–72.7 Ma for Asparagaceae crown age, (F4) 14.34–27.54 Ma for *Polygonatum* and *Heteropolygonatum*. The clock model uses the independent rate model (IRM), which follows a lognormal distribution. Nucleotide substitution selects the HKY model with alpha for gamma rates at sites set to 0.5. The birth–death process is used to generate uniform node age priors in the tree, using the default parameter (λ = 1, μ = 1, *s* = 0.1). The posterior probabilities of parameters were calculated using MCMC samples. The first 10% trees were discarded as burn-in and then sampled every 10 iterations until 20,000 samples were gathered.

## Results

### Sequencing, Assembly, and Annotation

**FIGURE 1:**
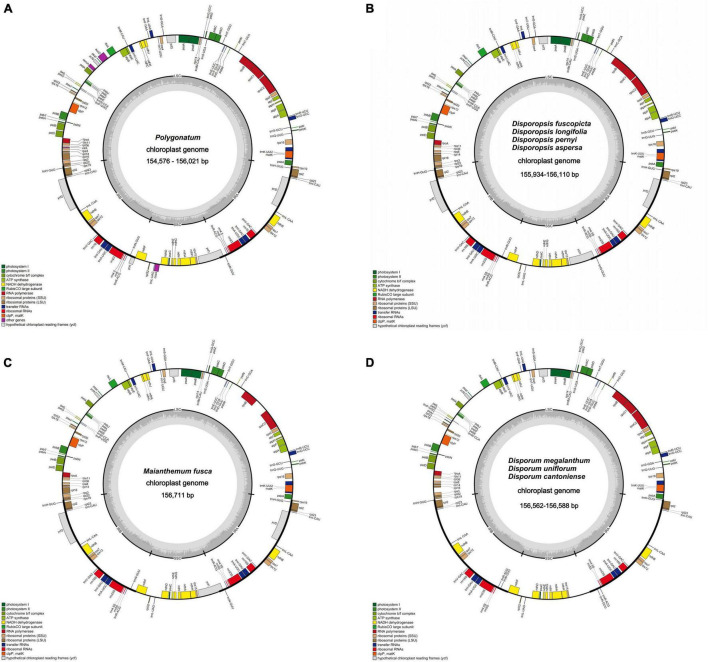
Chloroplast genome map: **(A)** *Polygonatum*, **(B)** *Disporopsis*, **(C)** *Maianthemum*, **(D)** *Disporum.* Genes inside and outside the circle are transcribed in a clockwise and counter-clockwise direction.

The raw data of 27 individuals were filtered to remove adapters and low-quality reads; 3–5 Gb data were obtained for each species in this study. After assembly and splicing, the complete cp genomes of the circular tetrad structure were obtained (Figure 1). The annotated result suggested that the cp genome length of *P. odoratum* (154,576 bp) was the smallest, and the cp genome length of *M. fuscum* (156,711 bp) was the largest among the 27 individuals. The length of the LSC region ranged from 83,493 bp (*P. odoratum*) to 85,218 bp (*M. fuscum*). The length of the SSC region ranged from 18,002 bp (*D. cantoniense*) to 18,573 bp (*P. cirrhifolium*), and the length of the IRa and IRb regions ranged from 26,242 bp (*D. fuscopicta*) to 26,815 bp (*D. cantoniense*). The GC content of the cp genomes ranged from 37.6 to 37.8% and varied among the different regions of the cp genomes. In addition, the number of genes and introns were highly conserved (Table 1), and the same suite of rRNA genes and tRNA genes was found in all taxa. All genomes have 85–88 protein-coding genes, except for *D. cantoniense, D. megalanthum*, and *D. uniflorum*, with 83 protein-coding genes (lacking *rps16* and *rpl32* genes) (Supplementary Table 4). It is worth noting that 19 genes were repeated in the *Polygonatum*, which were involved in photosynthesis and self-replication (Supplementary Table 3).

### Repeat Analysis

Repetitive sequences in the cp genome play a critical role in genome evolution and rearrangements. Oligonucleotide repeats analysis of four types of repeats in the cp genome, including Forward (F), Reverse (R), Palindromic (P), and Complementary (C), was performed by REPuter. The number of repeat types varied among the 26 cp genomes and presented random permutations, but most repeat sequences existed in 20–29 bp (Supplementary Figure 2). The abundance of F and P repeats was higher than that of R and C repeats (Supplementary Figure 2). The minimum number of repeats was found in *Disporum uniflorum* (60), whereas the maximum was found in *P. cirrhifolium*, *P. sibiricum*, and *P. zanlanscianense* (86). Complete details have been listed in Supplementary Tables 5, 6. Moreover, Spearman’s Rho correlation coefficients were obtained between tandem repeats, indels, and SNPs (Supplementary Table 7b). All these correlations showed a significant value (tandem repeats and indels: *p* < 0.001, tandem repeats and SNPs: *p* < 0.001, indels and SNPs: *p* < 0.001). The average correlation values between tandem repeats and indels, indels and SNPs, and tandem repeats in 26 *Polygonatum* species were 0.469, 0.351, and 0.267, respectively. Furthermore, we identified 67 (*P. franchetii*)–87 (*M. fuscum*) SSRs per cp genome consisting of mono- to hexa-nucleotide repeating units (Supplementary Table 8). Most of the SSRs were located in the intergenic areas. More than half of these SSRs (52.94–66.23%) were mononucleotide A/T motif, followed by dinucleotide (18.29–26.47%) with a predominant motif of AT/TA, tetranucleotide repeats (10.81–13.89%) with a predominant motif of AAAT/ATTT, AATC/ATTG, trinucleotide (2.94–6.94%), pentanucleotide (2.44–4.35%) with a predominant motif of AAACG/CGTTT, and hexanucleotides (0–1.35%) were absent in the cp genome of *M. fuscum* (Supplementary Figure 3).

### Inverted Repeats Regions Contraction and Expansion

**FIGURE 2:**
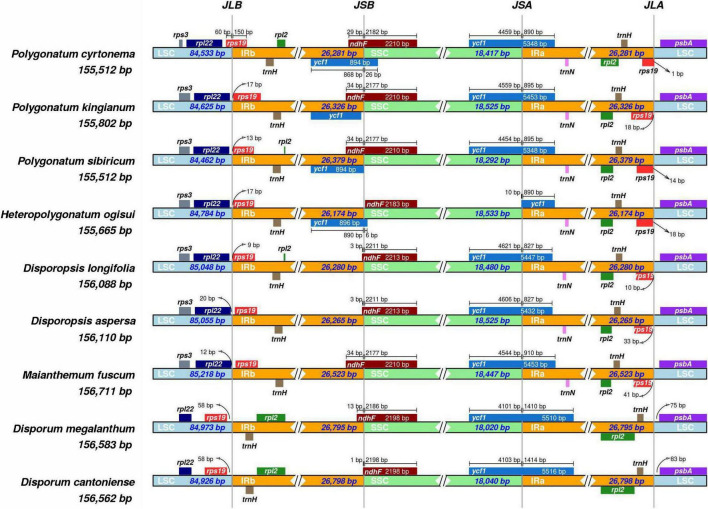
Comparisons of the borders of LSC, SSC, and IRa/b regions among the *Polygonatum*, *Heteropolygonatum*, *Disporopsis*, *Maianthemum*, and *Disporum.*

The contraction and expansion of IR regions revealed variation in LSC/IR/SSC regions (Figure 2). The *rpl22* gene was present in the LSC region, and *rpl2* and t*rnH* existed entirely in the IRb region. The *rps19* gene was present in the junction of the IRa/IRb/LSC region in four genera (*Polygonatum, Heteropolygonatum, Disporopsis*, and *Maianthemum*), but in *Disporum*, the *rps19* gene was absent in IRa/LSC region and existed completely in the IRb region. Notably, *rps19* was started in IRa regions and integrated into the LSC by 60 base pairs in *P. cyrtonema*, whereas in all other species of *Polygonatum*, the *rps19* gene exists completely in the LSC region. Additionally, the *ndhF* gene was observed at the junction of IRb/SSC and integrated into the SSC varied from 21 to 34 bp. Another truncated copy of the *ycf1* gene was observed in all species at the IRa/SSC junction, which starts in IRa regions and integrates into the SSC. In addition, *rpl2* and *trnH* existed in the IRa, and *psbA* existed in the LSC. It is worth noting that the *rps19* and *trnN* genes existed entirely in the IRa region of four genera, whereas the two genes were missing in the *Disporum* at the junction of IRa and LSC (Supplementary Figure 4). Moreover, the genome alignment analysis showed that the cp genomes among the 26 species were relatively conserved, and no inversions, translocations, and genomic rearrangements were detected (Supplementary Figures 5, 6).

### Comparative Chloroplast Genomic Analysis

**FIGURE 3:**
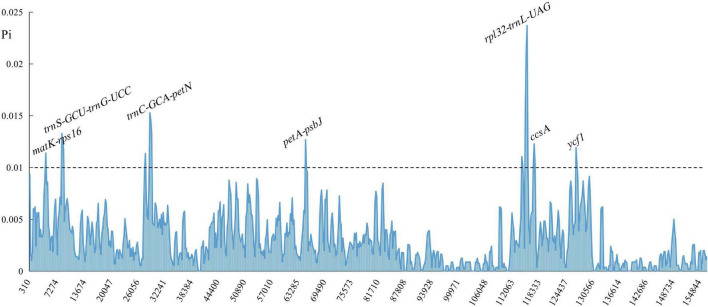
Sliding window analysis of *Polygonatum* cp genome. The X-axis represents the midpoint of the window; the Y-axis represents nucleotide diversity values.

Comparison of overall sequence variation showed that the cp genome within *Polygonatum* is highly conserved. The IR regions had lower sequence divergence than LSC and SSC regions. In addition, the coding region was more conserved than the non-coding regions. Furthermore, except for the more remarkable mutation in *ndhA, ycf1*, and *ycf2* genes, most of the protein-coding genes of *Polygonatum* were pretty conserved. The highest divergence in intergenic regions was found in the *rps16-trnQ-UUG, trnS-GCU-trnG-UCC, trnT-UGU-trnL-UAA, ndhC-trnV-UAC, rpl32-trnL-UAG, trnV-GAC-rps7*, and *accD-psaI*. The most divergent in the coding region were the *ycf1* and *ycf2* open reading frames (Supplementary Figure 7). Moreover, the sliding window analysis demonstrated that the seven regions had greater nucleotide diversity values (>0.01), including *matK-rps16, trnS-GCU-trnG-UCC, rpl32-trnL-UAG, trnC-GCA-petN, petA-psbJ, ccsA*, and *ycf1* (Figure 3). The polymorphism loci of these variability regions are listed in Supplementary Table 9. Among these regions, nucleotide diversity values of *rps16-trnQ-UUG, trnS-GCU-trnG-UCC, rpl32-trnL-UAG*, and *trnC-GCA-petN* were greater than 0.01, and the ycf1 gene was the lowest (0.00047). The insertions/deletions (indels) diversity of *trnS-GCU-trnG-UCC* and *matK-rps16* were 7.076 and 5.181, respectively, with no indel events detected in the *ccsA* gene. Furthermore, the cp genome of *Polygonatum, Heteropolygonatum, Disporopsis*, and *Maianthemum* is similar with an average similarity of 99% but different from that of *Disporum* with an average similarity of 85% based on the global comparison (Supplementary Figure 8).

### Phylogenetic Analysis

**FIGURE 4:**
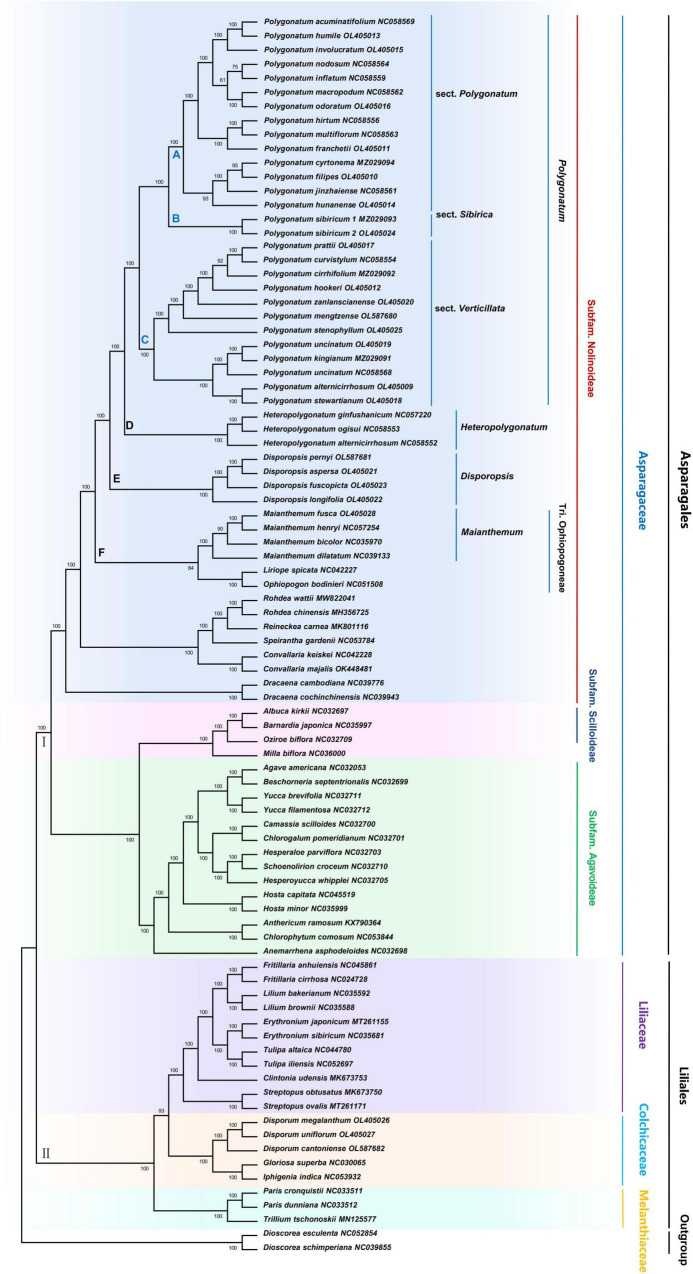
Maximum likelihood phylogenetic tree based on complete cp genome. *Dioscorea esculenta* and *D. schimperiana* were used as outgroups. Numbers at nodes are bootstrap support values.

The ML and NJ phylogenetic trees were inferred using 87 species, with the *Dioscorea* as the outgroup. The consensus trees obtained from the inference analyses were resolved, and most nodes were supported with maximum support (100% bootstrap support, Figure 4 and Supplementary Figure 9). The core Asparagaceae includes the subfamily of Scilloideae, Nolinoddeae, and Agavoideae, which form a monophyletic group (group I). Scilloideae and Agavoideae were sister taxa within the three subfamilies, and Nolinoddeae was a sister group to the clade of Scilloideae + Agavoideae. In addition, the results showed that most species of Trib. Polygonateae were placed in the crown of the phylogenetic tree, including *Polygonatum*, *Heteropolygonatu*m, and *Disporopsis*, and supported the monophyly of three genera. However, within this clade, the *Maianthemum* and Ophiopogoneae were sisters to a clade formed by *Disporopsis*, *Heteropolygonatum*, and *Polygonatum*, while *Disporum* was polyphyletic across three separate clades and distantly related to Trib. Polygonateae. In addition, the *Polygonatum* is further divided into sect. *Sibirica*, sect. *Polygonatum*, and sect. *Verticillata*. And the sister relationship was between sect. *Sibirica* and sect. *Polygonatum*, whereas sect. *Verticillata* was placed as sister to sect. *Polygonatum* + sect. *Sibirica* with high support (100% B/S). It is worth mentioning that sect. *Sibirica* only includes a species of *P. sibiricum.* The NJ and ML analyses produced trees with similar topologies, although some poorly supported groups were sensitive to changes in the mode of inference. The position of several species was unresolved, including *P. hunanense* and *P. kingianum*, which varied among trees recovered using distinct phylogenetic inference methods.

### Divergence Time Estimation

**FIGURE 5:**
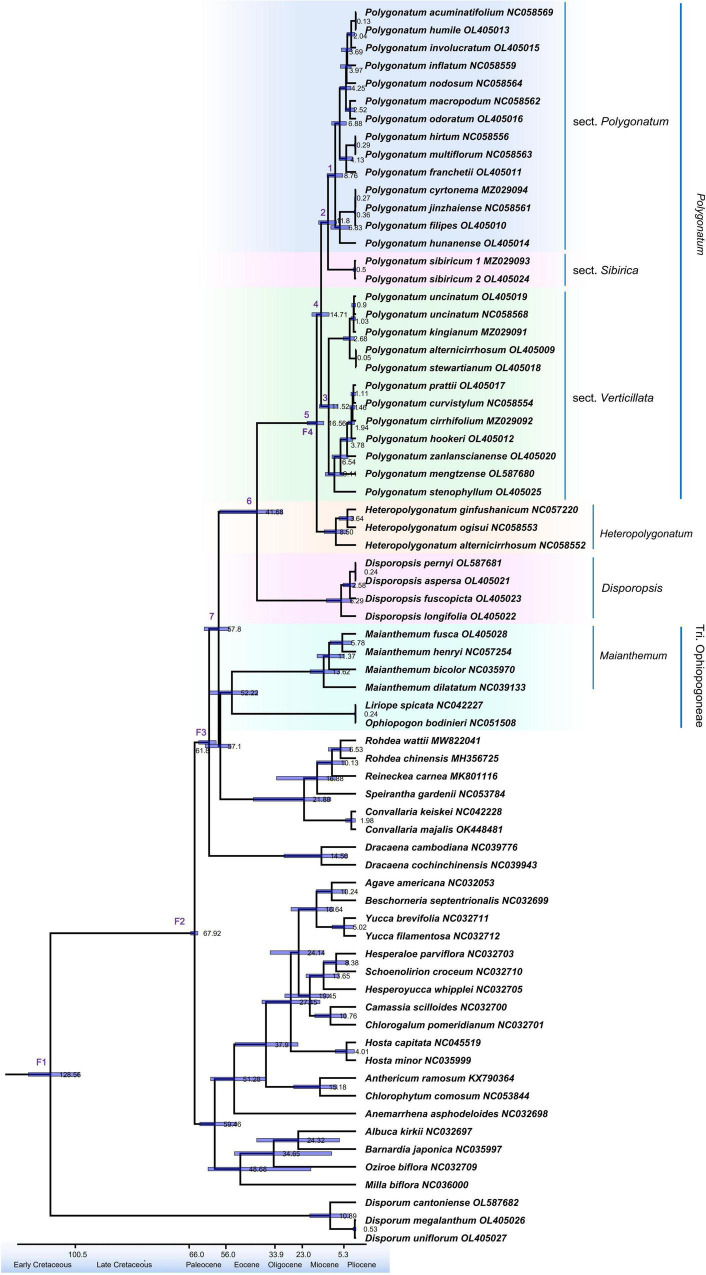
Divergence time estimation based on cp genome sequences. The divergence times are exhibited on each node, whereas the blue bars represent the 95% highest posterior density interval for each node age.

Results of divergence time for the node of the 95% highest posterior density (HPD) intervals are shown in Supplementary Table 10. A complete chronogram is demonstrated in Figure 5. The extant genera of the *Polygonatum* and *Disporopsis* have shared a common ancestor at the beginning of the Eocene (41.68 Ma, 31.97–57.29, 95% HPD), while the split between *Polygonatum* and *Heteropolygonatum* is estimated to occur at 16.56 Ma (HPD = 13.57–20.56 Ma, 95%), and sect. *Verticillata*, sect. *Polygonatum* and sect. *Sibirica* might share a common ancestor at 14.71 Ma (1.32–18.57 Ma, 95% HPD), and the divergence times between sect. *Polygonatum* and sect. *Sibirica* was formed at approximately 11.80 Ma. Sampled specimens of *Maianthemum* and *Ophiopogon* were estimated to have originated in 52.22 Ma. Moreover, the divergence times of the *Disporum* occurred at 128.56 Ma, having shared a common ancestor with the Asparagaceae.

### Reconstruction of Leaf Morphological Character

**FIGURE 6:**
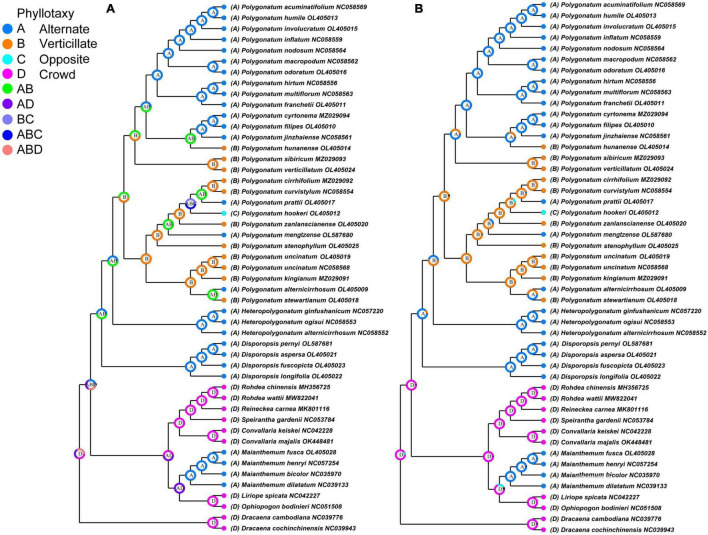
Ancestral state reconstructions of leaf arrangement in genus Polygonatum. **(A)** S-DIVA method and **(B)** MRBT method.

In the classification of *Polygonatum*, the arrangement of leaves was usually concerned. Phyllotaxy is one of the main characteristics of *Polygonatum* taxonomy, character transformation of leaf order is essential to understanding the evolution of *Polygonatum*. The phyllotaxy was used to reconstruct ancestral traits of *Polygonatum* and its relative species. As illustrated in Figure 6, the S-DIVA results showed that the verticillate leaves arrangement was the most likely ancestral state of *Polygonatum*, which was consistent with the MRBT method (B: *p* = 0.95). In addition, sect. *Polygonatum* is marked by phyllotaxy with an alternate leaf, except for *P. hunanense*. In its sister clade, sect. *Sibirica* is mostly a verticillate leaf arrangement. Sect. *Verticillata* includes species that appear to have a combination of the opposite, alternate, and verticillate leaves. In addition, alternate and verticillate leaves evolved more than once. Notably, the *Heteropolygonatum* and *Disporopsis* showed alternative phyllotaxy.

## Discussion

### Chloroplast Genome Structure and Comparative Analysis

In the present study, we *de novo* assembled the cp genome of 26 species of Trib. Polygonateae and performed comparative analyses. The cp genome of 26 species exhibited a quadripartite structure with two IR regions separated by the LSC and SSC regions. Higher GC content was observed in the IR region compared with the LSC and SSC regions, consistent with previous reports (Liang et al., 2021). In addition, our results found that the total length, GC content, and gene composition of the cp genome were almost identical in all species. Previous studies have found that the angiosperms possessed a highly conserved nature in the cp genome at the genus level (Yu et al., 2019; Shahzadi et al., 2020), but we found that the *rps16* and *rpl32* genes lost in *Disporum*, and this variation may be specific to *Disporum*.

Inverted repeat contraction and expansion could cause gene duplication, the origination of pseudogenes, and length variation in the cp genome, which were considered critical evolutionary phenomena. In the present study, *rps19* is present in the IR region in four genera (*Polygonatum, Heteropolygonatum, Disporopsis*, and *Maianthemum*), except for *Disporum*. Previous studies of monocotyledons’ cp genome revealed that the *rps19* gene existed in the IR region (Ahmed et al., 2012; Nock et al., 2014; Henriquez et al., 2020). Whereas the *de novo* assembled genome of *Disporum* contrast with previous studies, revealing integration of *rps19* into the LSC region. Moreover, the *ycf1* gene was duplicated in the IRa and IRb regions in *Polygonatum* and *Heteropolygonatum*, while in the other three genera (*Disporopsis, Maianthemum*, and *Disporum*), this gene only exists in the junction of the SSC/IRa region, which is consistent with a previous study (Wang et al., 2011).

At the genus level, weak-to-strong correlations among tandem repeats, SNPs, and indels have been observed in the *Polygonatum*. We found a weak correlation between tandem repeats and SNPs, a moderate correlation between indels and SNPs, and a strong correlation between tandem repeats and indels in *Polygonatum* plastomes. A recent study has confirmed that the plastomes exhibited strong associations between tandem repeats, indels, and substitutions in Araceae and Malvaceae (Abdullah et al., 2020, 2021). Our results also supported prior findings that tandem repeats play an important role in generating the indels and SNPs. These results have practical implications in selecting appropriate loci for comparative analyses.

### Phylogenetic and Taxonomic Resolution

The phylogeny and classification of *Polygonatum* have long been debated (Feng et al., 2020). This study used 88 cp genome, including most of the basal monocot family Asparagaceae, to construct the phylogenetic tree. Results of the NJ and ML phylogenies analysis confirmed the position of *Polygonatum* within the Asparagaceae, which were congruent and largely concordant with recent phylogenomic studies (Zhao et al., 2019; Xia et al., 2022). There is strong support for the monophyly of many major clades of Asparagaceae, including *Polygonatum*, *Heteropolygonatum*, *Disporopsis, Maianthemum*, and *Rohdea*. In a previous study, the *Polygonatum* was subdivided into two sections: sect. *Polygonatum* and sect. *Verticillata* based on the *trnK* (Tamura et al., 1997), but in our phylogeny, the *Polygonatum* was recovered as monophyletic in NJ and ML analyses, which were divided into three sections: sect. *Sibirica*, sect. *Polygonatum*, and sect. *Verticillata*, and can be strongly supported as a sister relationship between (1) *Polygonatum* and *Heteropolygonatum*, and (2) sect. *Sibirica* and sect. *Polygonatum*, and (3) sect. *Verticillata* and sect. *Polygonatum* + sect. *Sibirica*, respectively, which is consistent with previous studies (Meng et al., 2014; Floden, 2017; Zhao et al., 2019; Xia et al., 2022).

Moreover, we found several interesting implications of phylogeny in this study. First, both NJ and ML analyses provided strong evidence for the monophyly of *P. sibiricum* in sect. *Sibirica*. These results were supported by the findings of other researchers based on *matK*, *trnK*, *rbcL*, *psbA-trnH*, and cp genome (Meng et al., 2014; Floden, 2017; Zhao et al., 2019; Xia et al., 2022). In contrast, the cp genome tree placed *P. sibiricum* nested within sect. *Verticillata* or sect. *Sibirica* in a previous study (Floden and Schilling, 2018). Therefore, additional phylogeny analysis using an enormous collection of *Polygonatum* should be performed in the future. Second, previous phylogenetic studies of *Polygonatum* based on the cp genome have shown that the *Heteropolygonatum alternicirrhosum* belongs to the *Heteropolygonatum* and strongly supported the monophyly of *Heteropolygonatum* (Xia et al., 2022). However, in our study, the ML and NJ tree which strongly supported *P. alternicirrhosum* was deeply nested within the *Polygonatum* and supported the monophyly of *Polygonatum* and *Heteropolygonatum.* Our analyses suggest that *Polygonatum* may be the sister to the *Heteropolygonatum*. In addition, another previous study also supports that the species belongs to *Polygonatum* (Chen and Tamura, 2000). Therefore, our results suggested that *P. alternicirrhosum* (OL405009) and *H. alternicirrhosum* (NC058552) should be considered as two species, in contrast with other recent estimations, mainly to the influential Floden’s (2014) study, which suggested that *P. alternicirrhosum* should be transferred to *Heteropolygonatum* based of morphological, cytological comparisons and molecular data. In addition, Zhao et al. (2019) indicated that the *P. alternicirrhosum* should be recovered within the *Polygonatum*. Another example is the *Polygonatum mengtzense*, which was considered most closely related to the *P. punctatum* (Floden, 2014). In this study, maximum likelihood analysis reveals that *P. mengtzense* was deeply nested within sect. *Verticillata*, which was supported by molecular and chromosome evidence (Floden, 2014; Xia et al., 2022). Finally, there is a significant divergence in the classification of Trib. Polygonateae. In the previous study, Kim et al. (2010) placed *Disporopsis* as a sister to *Polygonatum* + *Maianthemumin* in a Bayesian analysis. In the present study, the phylogenetic analyses corroborated the monophyly of *Polygonatum, Heteropolygonatum, Disporopsis, Maianthemum*, and *Disporum.* The *Disporum* is sister to Glorioseae and nested within the family Colchicaceae (more distantly related species), consistent with previous genetic studies (Tamura et al., 2013).

It is interesting to note that the *Maianthemum* was traditionally included with Trib. Polygonateae based on morphology (Chen and Tamura, 2000), and other research based on multiple plastid markers (*atpB*, *ndhF*, *rbcL*, *matK*, *psbA-trnH*, *trnC-petN*, *atpB-rbcL*, and *rps16*) also supports its placement in Trib. Polygonateae (Chen et al., 2013; Meng et al., 2014; Zhao et al., 2019). However, ML analysis in our study reveals that the *Maianthemum* was deeply nested within Trib. Ophiopogoneae rather than Trib. Polygonateae, which agrees with former studies based on multiple loci (e.g., *petA-psbJ*, *ETS*, *ITS*, and *rps10*; or *trnL-F*, *rps16*, *rpl16*, *psbA-trnH, rbcL, trnK, trnC-petN*, and *ITS*) (Floden, 2017; Floden and Schilling, 2018; Meng et al., 2021). Our results suggested that the Trib. Polygonateae should include only three genera (*Disporopsis, Heteropolygonatum*, and *Polygonatum*). Therefore, the results revealed that the convergent evolution of some traits may have misled previous relationships. Further phylogenetic analysis is needed within the *Maianthemum*.

### Diversification History and Leaf Arrangements

Results of divergence time estimates suggest that the elevated diversification rates of *Polygonatum* occurred from approximately 15–0.1 Ma during the late Miocene and early Pliocene. The two main lineages, sect. *Verticillata* and sect. *Sibirica* + sect. *Polygonatum* seem to have radiated since the mid-Miocene (sect. *Verticillata*: 11.52 Ma; sect. *Sibirica* + sect. *Polygonatum*: 11.80 Ma; Figure 5; Supplementary Table 10). Notably, the diversification rates of *Polygonatum* slowly increased during this period, attributed to uplifts of the Qinghai–Tibetan Plateau in the early Miocene (Xue et al., 2021). In addition to the aforementioned tectonic rearrangements and mountain formation in East Asia, the global climatic fluctuations and aridification that occurred in the Mid-Miocene Climatic Optimum (MMCO, 15–17 Ma; Zachos et al., 2001) also accelerated the diversification rates of *Polygonatum*. Global warming occurred at approximately 15 Ma (MMCO), followed by a gradual decrease in temperature (Zachos et al., 2001). These climatic changes might have influenced the plant diversification and promoted radiation of *Polygonatum* species.

Variation in phyllotaxy morphology represents an important character source for species delimitation. The phyllotaxy diversity (alternate, opposite, and verticillate) caused some confusion in classifying the *Polygonatum*. Our study showed the evolutionary trend of *Polygonatum* from verticillate leaves to alternate leaves, and this suggests that verticillate leaf is the ancestral state and agrees with the previous molecular studies of *Polygonatum* (Xia et al., 2022). It is noteworthy that the phyllotaxy of *Polygonatum* is an unstable character even for the same species. Therefore, phyllotaxy cannot be used as the unique taxonomic feature for classifying *Polygonatum*.

## Conclusion

In this study, the complete cp genome of 26 species of Trib. Polygonateae was *de novo* assembled from Illumina reads. In all of our analyses, these cp genomes were generally conserved and exhibited similar gene content and genomic structure. A total of 8 highly variable loci were identified across the *Polygonatum* cp genome, which could serve as potential markers for phylogenetic and population genetics studies. The monophyly of *Polygonatum* was confirmed, and phylogenetic analysis indicated that the genus consists of three sections (sect. *Sibirica*, sect. *Polygonatum*, and sect. *Verticillata*). Meanwhile, the phylogenetic analysis suggested that *Heteropolygonatum* may be the sister group of the *Polygonatum*, but the *Disporopsis*, *Maianthemum*, and *Disporum* may have diverged earlier. In conclusion, our results enhanced the genomic information for *Polygonatum* and provided valuable insight into the phylogenetic relationships among the genera involved in Trib. Polygonateae. The results also contribute to the bioprospecting and conservation of the *Polygonatum*.

## Data Availability Statement

The datasets presented in this study can be found in online repositories. The names of the repository/repositories and accession number(s) can be found in the article/Supplementary Material.

## Author Contributions

JW, ZX, SC, and BD participated in the conception and design of the research. JW, JQ, FY, YJ, and BZ collected and identified the species. JW, JQ, and XC were responsible for analyzing and processing data. JW wrote the manuscript. ZX and BD revised the manuscript. All authors agreed to the submitted version of the manuscript.

## Conflict of Interest

The authors declare that the research was conducted in the absence of any commercial or financial relationships that could be construed as a potential conflict of interest.

## Publisher’s Note

All claims expressed in this article are solely those of the authors and do not necessarily represent those of their affiliated organizations, or those of the publisher, the editors and the reviewers. Any product that may be evaluated in this article, or claim that may be made by its manufacturer, is not guaranteed or endorsed by the publisher.

## Footnotes

1
https://bibiserv.cebitec.uni-bielefeld.de/reputer/

2
http://www.rub.de/ecoevo/cm/cm_phobos.htm

3
http://pgrc.ipk-gatersleben.de/misa/

4
https://irscope.shinyapps.io/irapp/
