# Supplementary material for: Comparative Analysis of Chloroplast Genome and New Insights Into Phylogenetic Relationships of Polygonatum and Tribe Polygonateae
Source: Front Plant Sci. 2022 Jun 24;13:882189. doi: 10.3389/fpls.2022.882189 (PMC9263837; doi:10.3389/fpls.2022.882189)
Supplement: Supplementary file 1 [file Table_1.pdf]

Table S1 Information about the samples collected.

| No. | Species | Cultivation location | Collection location | Voucher specimen |
|---|---|---|---|---|
| 1 | *Polygonatum kingianum* | Dali, Yunnan, China | Dali, Yunnan, China | HJ202001 |
| 2 | *P. cirrhifolium* | Dali, Yunnan, China | Dali, Yunnan, China | HJ202002 |
| 3 | *P. sibiricum 1* | Dali, Yunnan, China | Luoyang, Henan, China | HJ202003 |
| 4 | *P. cyrtonema* | Dali, Yunnan, China | Yichun, Jiangxi, China | HJ202004 |
| 5 | *P. alternicirrhosum* | Dali, Yunnan, China | Luding, Sichuan, China | HJ202122 |
| 6 | *P. stewartianum* | Dali, Yunnan, China | Yongping, Yunnan, China | HJ202123 |
| 7 | *P. hunanense* | Dali, Yunnan, China | Zhangjiajie, Hunan, China | HJ202124 |
| 8 | *P. uncinatum* | Dali, Yunnan, China | Dali, Yunnan, China | HJ202105 |
| 9 | *P. odoratum* | Dali, Yunnan, China | Qingyuan, Guandong, China | HJ202106 |
| 10 | *P. hookeri* | Dali, Yunnan, China | Lijiang, Yunnan, China | HJ202107 |
| 11 | *P. prattii* | Dali, Yunnan, China | Lijiang, Yunnan, China | HJ202108 |
| 13 | *P. filipes* | Dali, Yunnan, China | Wenzhou, Zhejiang, China | HJ202109 |
| 12 | *P. mengtzense* | Enshi, Hubei, China | Wenshan, Yunnan, China | HJ202110 |
| 14 | *P. franchetii* | Enshi, Hubei, China | Dehong, Yunnan, China | HJ202111 |
| 15 | *P. sibiricum 2* | Enshi, Hubei, China | Yongping, Yunnan, China | HJ202112 |
| 16 | *P. zanlanscianense* | Enshi, Hubei, China | Yichang, Hubei, China | HJ202113 |
| 17 | *P. humile* | Dali, Yunnan, China | Harbin, Heilongjiang, China | HJ202125 |
| 18 | *P. involucratum* | Dali, Yunnan, China | Harbin, Heilongjiang, China | HJ202126 |
| 19 | *P. stenophyllum* | Dali, Yunnan, China | Harbin, Heilongjiang, China | HJ202127 |
| 20 | *Disporopsis aspersa* | Enshi, Hubei, China | Laibing, Guangxi, China | ZGQ202114 |
| 21 | *Disporopsis longifolia* | Enshi, Hubei, China | Guilin, Guangxi, China | ZGQ202115 |
| 22 | *Disporopsis fuscopicta* | Enshi, Hubei, China | Laibing, Guangxi, China | ZGQ202116 |
| 23 | *Disporopsis pernyi* | Enshi, Hubei, China | Laibing, Guangxi, China | ZGQ202117 |
| 24 | *Disporum megalanthum* | Enshi, Hubei, China | Enshi, Hubei, China | WSZ202118 |
| 25 | *Disporum uniflorum* | Enshi, Hubei, China | Enshi, Hubei, China | WSZ202119 |
| 26 | *Disporum cantoniense* | Enshi, Hubei, China | Enshi, Hubei, China | WSZ202120 |
| 27 | *Maianthemum fuscum* | Enshi, Hubei, China | Dehong, Yunnan, China | WHC202121 |

Table S2 Species information downloaded by NCBI.

| No. | Species | GenBank accession | No. | Species | GenBank accession |
|---|---|---|---|---|---|
| 1 | *P. acuminatifolium* | NC058569 | 32 | *Yucca brevifolia* | NC032711 |
| 2 | *P. nodosum* | NC058564 | 33 | *Yucca filamentosa* | NC032712 |
| 3 | *P. inflatum* | NC058559 | 34 | *Camassia scilloides* | NC032700 |
| 4 | *P. macropodum* | NC058562 | 35 | *Chlorogalum pomeridianum* | NC032701 |
| 5 | *P. hirtum* | NC058556 | 36 | *Hesperaloe parviflora* | NC032703 |
| 6 | *P. multiflorum* | NC058563 | 37 | *Schoenolirion croceum* | NC032710 |
| 7 | *P. jinzhaiense* | NC058561 | 38 | *Hesperoyucca whipplei* | NC032705 |
| 8 | *P. curvistylum* | NC058554 | 39 | *Hosta capitata* | NC045519 |
| 9 | *P. uncinatum* | NC058568 | 40 | *Hosta minor* | NC035999 |
| 10 | *Heteropolygonatum ginfushanicum* | NC057220 | 41 | *Anthericum ramosum* | KX790364 |
| 11 | *Heteropolygonatum ogisui* | NC058553 | 42 | *Chlorophytum comosum* | NC053844 |
| 12 | *Heteropolygonatum alternicirrhosum* | NC058552 | 43 | *Anemarrhena asphodeloides* | NC032698 |
| 13 | *Maianthemum henryi* | NC057254 | 44 | *Fritillaria anhuiensis* | NC045861 |
| 14 | *Maianthemum bicolor* | NC035970 | 45 | *Fritillaria cirrhosa* | NC024728 |
| 15 | *Maianthemum dilatatum* | NC039133 | 46 | *Lilium bakerianum* | NC035592 |
| 16 | *Liriope spicata* | NC042227 | 47 | *Lilium brownii* | NC035588 |
| 17 | *Ophiopogon bodinieri* | NC051508 | 48 | *Erythronium japonicum* | MT261155 |
| 18 | *Rohdea wattii* | MW822041 | 49 | *Erythronium sibiricum* | NC035681 |
| 19 | *Rohdea chinensis* | MH356725 | 50 | *Tulipa altaica* | NC044780 |
| 20 | *Reineckea carnea* | MK801116 | 51 | *Tulipa iliensis* | NC052697 |
| 21 | *Speirantha gardenii* | NC053784 | 52 | *Clintonia udensis* | MK673753 |
| 22 | *Convallaria keiskei* | NC042228 | 53 | *Streptopus obtusatus* | MK673750 |
| 23 | *Convallaria majalis* | OK448481 | 54 | *Streptopus ovalis* | MT261171 |
| 24 | *Dracaena cambodiana* | NC039776 | 55 | *Paris cronquistii* | NC033511 |
| 25 | *Dracaena cochinchinensis* | NC039943 | 56 | *Paris dunniana* | NC033512 |
| 26 | *Albuca kirkii* | NC032697 | 57 | *Trillium tschonoskii* | MN125577 |
| 27 | *Barnardia japonica* | NC035997 | 58 | *Gloriosa superba* | NC030065 |
| 28 | *Oziroe biflora* | NC032709 | 59 | *Iphigenia indica* | NC053932 |
| 29 | *Milla biflora* | NC036000 | 60 | *Dioscorea esculenta* | NC052854 |
| 30 | *Agave americana* | NC032053 | 61 | *Dioscorea schimperiana* | NC039855 |
| 31 | *Beschorneria septentrionalis* | NC032699 | | | |

Table S3 Gene composition in *Polygonatum* chloroplast genome.

| Category for genes | Group of genes | Name of genes |
|---|---|---|
| Self replication | Ribosomal RNAs（rRNA） | *rrn16S*, rrn23S*, rrn4.5S*, rrn5S** |
| | Transfer RNAs（tRNA） | *trnK-UUU, trnQ-UUG, trnS-GCU, trnG-UCC, trnR-UCU, trnC-GCA, trnD-GUC, trnY-GUA, trnE-UUC, trnT-GGU, trnS-UGA, trnG-UCC, trnfM-CAU*, trnS-GGA, trnT-UGU, trnL-UAA, trnF-GAA, trnV-UAC, trnW-CCA, trnP-UGG, trnH-GUG*, trnI-CAU*, trnL-CAA*, trnV-GAC*, trnI-GAU*, trnA-UGC*, trnR-ACG*, trnN-GUU*, trnL-UAG* |
| | Small subunit of ribosome | *rps11, rps12*, rps14, rps15, rps16, rps18, rps19*, rps2, rps3, rps4, rps7*, rps8* |
| | Large subunit of ribosome | *rpl14, rpl16, rpl2*, rpl20, rpl22, rpl23*, rpl32, rpl33, rpl36* |
| | RNA polymerase | *rpoA, rpoB, rpoC1, rpoC2* |
| Genes for Photosynthesis | PhotosystemI | *psaA, psaB, psaC, psaI, psaJ* |
| | PhotosystemII | *psbA, psbB, psbC, psbD, psbE, psbF, psbI, psbJ, psbK, psbL, psbM, psbN, psbT, psbZ* |
| | Cytochrome b/f complex | *petA, petB, petD, petG, petL, petN* |
| | ATP synthase | *aptA, aptB, aptF, aptH, aptI* |
| | Rubisco | *rbcL* |
| | NADH dehydrogenase | *ndhA, ndhB*, ndhC, ndhD, ndhE, ndhF, ndhG, ndhH, ndhI, ndhJ, ndhK* |
| Other genes | Maturase | *matK* |
| | Envelop membrane protein | *cemA* |
| | acetyl-CoA-carboxylase | *accD* |
| | c-type cytochrome synthesis | *ccsA* |
| | Protease | *clpP* |
| Genes of unknown function | Conserved open reading Frames | *Ycf1, ycf2*, ycf3, ycf4* |

* indicates genes duplicated in the chloroplast genome.

Table S4 Gene composition in *Disporum* chloroplast genome.

| Category for genes | Group of genes | Name of genes |
|---|---|---|
| Self replication | Ribosomal RNAs（rRNA） | *rrn16S*, rrn23S*, rrn4.5S*, rrn5S** |
| | Transfer RNAs（tRNA） | *trnK-UUU, trnQ-UUG, trnS-GCU, trnG-UCC, trnR-UCU, trnC-GCA, trnD-GUC, trnY-GUA, trnE-UUC, trnT-GGU, trnS-UGA, trnG-UCC, trnfM-CAU*, trnS-GGA, trnT-UGU, trnL-UAA, trnF-GAA, trnV-UAC, trnW-CCA, trnP-UGG, trnH-GUG*, trnI-CAU*, trnL-CAA*, trnV-GAC*, trnI-GAU*, trnA-UGC*, trnR-ACG*, trnN-GUU*, trnL-UAG* |
| | Small subunit of ribosome | *rps11, rps12*, rps14, rps15, rps18, rps19*, rps2, rps3, rps4, rps7*, rps8* |
| | Large subunit of ribosome | *rpl14, rpl16, rpl2*, rpl20, rpl22, rpl23*, rpl33, rpl36* |
| | RNA polymerase | *rpoA, rpoB, rpoC1, rpoC2* |
| Genes for Photosynthesis | PhotosystemI | *psaA, psaB, psaC, psaI, psaJ* |
| | PhotosystemII | *psbA, psbB, psbC, psbD, psbE, psbF, psbI, psbJ, psbK, psbL, psbM, psbN, psbT, psbZ* |
| | Cytochrome b/f complex | *petA, petB, petD, petG, petL, petN* |
| | ATP synthase | *aptA, aptB, atpE,, aptF, aptH, aptI* |
| | Rubisco | *rbcL* |
| | NADH dehydrogenase | *ndhA, ndhB*, ndhC, ndhD, ndhE, ndhF, ndhG, ndhH, ndhI, ndhJ, ndhK* |
| Other genes | Maturase | *matK* |
| | Envelop membrane protein | *cemA* |
| | acetyl-CoA-carboxylase | *accD* |
| | c-type cytochrome synthesis | *ccsA* |
| | Protease | *clpP* |
| Genes of unknown function | Conserved open reading Frames | *ycf1, ycf2, ycf4* |

* indicates genes duplicated in the chloroplast genome.

Table S5 The number of reverse repeats (R) and palindromic repeats (P) in the cp genome.

| Species | R | | | | | | | P | | | | | | |
|---|---|---|---|---|---|---|---|---|---|---|---|---|---|---|
| | 20-29 | 30-39 | 40-49 | 50-59 | 60-69 | ≥70 | Total | 20-29 | 30-39 | 40-49 | 50-59 | 60-69 | ≥70 | Total |
| *P. kingianum* | 14 | 1 | 0 | 0 | 0 | 0 | 15 | 32 | 9 | 2 | 3 | 0 | 0 | 46 |
| *P. cirrhifolium* | 6 | 1 | 0 | 0 | 0 | 0 | 7 | 27 | 15 | 2 | 3 | 0 | 2 | 49 |
| *P. sibiricum 1* | 9 | 1 | 0 | 0 | 0 | 0 | 10 | 27 | 14 | 2 | 1 | 0 | 2 | 46 |
| *P. cyrtonema* | 8 | 1 | 0 | 0 | 0 | 0 | 9 | 37 | 9 | 0 | 3 | 2 | 0 | 51 |
| *P. alternicirrhosum* | 8 | 1 | 0 | 0 | 0 | 0 | 9 | 30 | 13 | 4 | 3 | 0 | 0 | 50 |
| *P. filipes* | 8 | 1 | 0 | 0 | 0 | 0 | 9 | 36 | 9 | 0 | 3 | 2 | 0 | 50 |
| *P. franchetii* | 12 | 1 | 0 | 0 | 0 | 0 | 13 | 32 | 12 | 2 | 1 | 0 | 0 | 47 |
| *P. hookeri* | 7 | 1 | 0 | 0 | 0 | 0 | 8 | 28 | 15 | 2 | 3 | 0 | 2 | 50 |
| *P. humile* | 9 | 1 | 0 | 0 | 0 | 0 | 10 | 34 | 12 | 2 | 3 | 2 | 0 | 53 |
| *P. hunanense* | 8 | 2 | 0 | 0 | 0 | 0 | 10 | 35 | 9 | 1 | 3 | 2 | 0 | 50 |
| *P. involucratum* | 12 | 1 | 0 | 0 | 0 | 0 | 13 | 28 | 12 | 2 | 3 | 2 | 0 | 47 |
| *P. odoratum* | 12 | 1 | 0 | 1 | 0 | 0 | 14 | 31 | 13 | 2 | 1 | 0 | 0 | 47 |
| *P. prattii* | 8 | 1 | 0 | 1 | 0 | 0 | 10 | 28 | 14 | 2 | 3 | 0 | 2 | 49 |
| *P. mengtzense* | 14 | 2 | 0 | 0 | 0 | 0 | 16 | 26 | 10 | 2 | 3 | 2 | 0 | 43 |
| *P. stewartianum* | 9 | 1 | 0 | 0 | 0 | 0 | 10 | 29 | 13 | 4 | 3 | 0 | 0 | 49 |
| *P. uncinatum* | 13 | 2 | 0 | 0 | 0 | 0 | 15 | 36 | 9 | 0 | 1 | 0 | 2 | 48 |
| *P. sibiricum 2* | 9 | 1 | 0 | 0 | 0 | 0 | 10 | 27 | 14 | 2 | 1 | 0 | 0 | 44 |
| *P. zanlanscianense* | 6 | 1 | 0 | 0 | 0 | 0 | 7 | 25 | 15 | 4 | 3 | 0 | 2 | 49 |
| *P. stenophyllum* | 13 | 0 | 0 | 0 | 0 | 0 | 13 | 31 | 9 | 4 | 3 | 0 | 0 | 47 |
| *Disporopsis aspersa* | 13 | 1 | 0 | 0 | 0 | 0 | 14 | 25 | 11 | 2 | 4 | 0 | 0 | 42 |
| *Disporopsis longifolia* | 15 | 2 | 0 | 0 | 0 | 0 | 17 | 32 | 10 | 4 | 1 | 0 | 0 | 47 |
| *Disporopsis fuscopicta* | 14 | 1 | 0 | 0 | 0 | 0 | 15 | 26 | 13 | 2 | 2 | 0 | 2 | 45 |
| *Disporopsis pernyi* | 12 | 1 | 0 | 0 | 0 | 0 | 13 | 25 | 13 | 2 | 2 | 0 | 2 | 44 |
| *Disporum megalanthum* | 18 | 0 | 0 | 0 | 0 | 0 | 18 | 36 | 9 | 2 | 1 | 1 | 0 | 49 |
| *Disporum uniflorum* | 19 | 0 | 0 | 0 | 0 | 0 | 19 | 37 | 9 | 2 | 1 | 1 | 0 | 50 |
| *Disporum cantoniense* | 23 | 0 | 0 | 0 | 0 | 0 | 23 | 33 | 9 | 2 | 0 | 0 | 1 | 45 |
| *Maianthemum fuscum* | 14 | 2 | 0 | 0 | 0 | 0 | 16 | 32 | 13 | 2 | 1 | 0 | 0 | 48 |

Table S6 The number of fowarde (F) and complementary repeats (C) in the cp genome.

| Species | F | | | | | | | C | | | | | | |
|---|---|---|---|---|---|---|---|---|---|---|---|---|---|---|
| | 20-29 | 30-39 | 40-49 | 50-59 | 60-69 | ⩾70 | Total | 20-29 | 30-39 | 40-49 | 50-59 | 60-69 | ⩾70 | Total |
| *P. kingianum* | 23 | 4 | 2 | 2 | 1 | 1 | 33 | 5 | 0 | 0 | 0 | 0 | 0 | 5 |
| *P. cirrhifolium* | 20 | 9 | 4 | 3 | 2 | 3 | 41 | 1 | 1 | 0 | 0 | 0 | 0 | 2 |
| *P. sibiricum 1* | 22 | 7 | 4 | 1 | 2 | 3 | 39 | 4 | 0 | 0 | 0 | 0 | 0 | 4 |
| *P. cyrtonema* | 23 | 6 | 1 | 1 | 3 | 1 | 35 | 4 | 0 | 0 | 0 | 0 | 0 | 4 |
| *P. alternicirrhosum* | 20 | 6 | 5 | 2 | 1 | 1 | 35 | 5 | 0 | 0 | 0 | 0 | 0 | 5 |
| *P. filipes* | 23 | 5 | 1 | 1 | 3 | 1 | 34 | 6 | 0 | 0 | 0 | 0 | 0 | 6 |
| *P. franchetii* | 23 | 6 | 3 | 0 | 1 | 1 | 34 | 5 | 0 | 0 | 0 | 0 | 0 | 5 |
| *P. hookeri* | 20 | 8 | 4 | 3 | 2 | 3 | 40 | 1 | 0 | 0 | 0 | 0 | 0 | 1 |
| *P. humile* | 19 | 5 | 3 | 1 | 3 | 1 | 32 | 4 | 0 | 0 | 0 | 0 | 0 | 4 |
| *P. hunanense* | 23 | 5 | 2 | 1 | 3 | 1 | 35 | 4 | 0 | 0 | 0 | 0 | 0 | 4 |
| *P. involucratum* | 22 | 5 | 3 | 1 | 3 | 1 | 35 | 4 | 0 | 0 | 3 | 0 | 0 | 7 |
| *P. odoratum* | 25 | 5 | 3 | 0 | 1 | 1 | 35 | 4 | 0 | 0 | 1 | 0 | 0 | 5 |
| *P. prattii* | 20 | 8 | 4 | 3 | 2 | 3 | 40 | 1 | 0 | 0 | 1 | 0 | 0 | 2 |
| *P. mengtzense* | 22 | 3 | 3 | 1 | 3 | 1 | 33 | 7 | 0 | 0 | 0 | 0 | 0 | 7 |
| *P. stewartianum* | 20 | 6 | 5 | 2 | 1 | 1 | 35 | 5 | 0 | 0 | 0 | 0 | 0 | 5 |
| *P. uncinatum* | 24 | 4 | 1 | 2 | 1 | 1 | 33 | 7 | 0 | 0 | 0 | 0 | 0 | 7 |
| *P. sibiricum 2* | 22 | 6 | 4 | 1 | 2 | 3 | 38 | 4 | 0 | 0 | 0 | 0 | 0 | 4 |
| *P. zanlanscianense* | 20 | 7 | 7 | 3 | 2 | 3 | 42 | 1 | 0 | 0 | 0 | 0 | 0 | 1 |
| *P. stenophyllum* | 23 | 5 | 5 | 2 | 1 | 1 | 37 | 2 | 0 | 0 | 0 | 0 | 0 | 2 |
| *Disporopsis aspersa* | 17 | 6 | 5 | 3 | 2 | 3 | 36 | 5 | 0 | 0 | 0 | 0 | 0 | 5 |
| *Disporopsis longifolia* | 21 | 4 | 4 | 0 | 1 | 1 | 31 | 4 | 0 | 0 | 0 | 0 | 0 | 4 |
| *Disporopsis fuscopicta* | 15 | 7 | 4 | 1 | 2 | 3 | 32 | 6 | 1 | 0 | 0 | 0 | 0 | 7 |
| *Disporopsis pernyi* | 19 | 7 | 4 | 1 | 2 | 0 | 33 | 6 | 0 | 0 | 0 | 0 | 0 | 6 |
| *Disporum megalanthum* | 18 | 5 | 2 | 0 | 0 | 0 | 25 | 7 | 0 | 0 | 0 | 0 | 0 | 7 |
| *Disporum uniflorum* | 17 | 5 | 2 | 0 | 0 | 0 | 24 | 6 | 0 | 0 | 0 | 0 | 0 | 6 |
| *Disporum cantoniense* | 18 | 5 | 2 | 0 | 0 | 0 | 25 | 6 | 0 | 0 | 0 | 0 | 0 | 6 |
| *Maianthemum fuscum* | 17 | 6 | 4 | 0 | 1 | 1 | 29 | 6 | 0 | 0 | 0 | 0 | 0 | 6 |

Table S7b Spearman's Rho correlation analysis result among tandem repeats, indels and SNPs using Minitab based on plastome alignments between *Polygonatum* species presented here with *P. sibiricum*_1 as a reference (150 bp windows).

| | Tandem repeats and SNPs | Indels and SNPs | Tandem repeats and Indels |
|---|---|---|---|
| Rho | 0.267 | 0.351 | 0.469 |
| P-value | 0.000*** | 0.000*** | 0.000*** |

***Correlation was strongly significant at p<0.001

Table S8 Number of SSR types in the chloroplast genome.

| Species | mono- | di- | tri- | tetra- | penta- | hexa- | Total |
|---|---|---|---|---|---|---|---|
| *P. kingianum* | 42 | 16 | 5 | 10 | 2 | 0 | 75 |
| *P. cirrhifolium* | 41 | 16 | 3 | 8 | 2 | 0 | 70 |
| *P. sibiricum 1* | 36 | 18 | 3 | 9 | 2 | 0 | 68 |
| *P. cyrtonema* | 42 | 16 | 5 | 8 | 2 | 1 | 74 |
| *P. alternicirrhosum* | 40 | 15 | 5 | 10 | 2 | 0 | 72 |
| *P. filipes* | 42 | 16 | 5 | 8 | 2 | 1 | 74 |
| *P. franchetii* | 39 | 15 | 3 | 8 | 2 | 0 | 67 |
| *P. hookeri* | 40 | 16 | 3 | 8 | 2 | 0 | 69 |
| *P. humile* | 44 | 15 | 5 | 8 | 2 | 0 | 74 |
| *P. hunanense* | 39 | 16 | 3 | 8 | 3 | 0 | 69 |
| *P. involucratum* | 43 | 16 | 4 | 8 | 2 | 0 | 73 |
| *P. odoratum* | 44 | 15 | 4 | 8 | 2 | 0 | 73 |
| *P. prattii* | 42 | 15 | 3 | 8 | 2 | 0 | 70 |
| *P. mengtzense* | 52 | 15 | 4 | 9 | 2 | 0 | 82 |
| *P. stewartianum* | 40 | 15 | 5 | 10 | 2 | 0 | 72 |
| *P. uncinatum* | 43 | 16 | 5 | 10 | 2 | 0 | 76 |
| *P. sibiricum 2* | 40 | 16 | 3 | 9 | 2 | 0 | 70 |
| *P. zanlanscianense* | 42 | 15 | 3 | 8 | 3 | 0 | 71 |
| *P. stenophyllum* | 42 | 16 | 3 | 9 | 2 | 0 | 72 |
| *Disporopsis aspersa* | 51 | 15 | 4 | 9 | 2 | 0 | 81 |
| *Disporopsis longifolia* | 49 | 15 | 4 | 10 | 2 | 1 | 81 |
| *Disporopsis fuscopicta* | 41 | 16 | 5 | 9 | 2 | 1 | 74 |
| *Disporopsis pernyi* | 51 | 9 | 7 | 9 | 0 | 1 | 77 |
| *Disporum megalanthum* | 55 | 9 | 6 | 10 | 0 | 1 | 81 |
| *Disporum uniflorum* | 54 | 9 | 6 | 10 | 0 | 1 | 80 |
| *Disporum cantoniense* | 45 | 14 | 3 | 9 | 2 | 0 | 73 |
| *Maianthemum fuscum* | 57 | 16 | 2 | 9 | 3 | 0 | 87 |

Table S9 Identified polymorphic loci based on comparative plastome analysis of *Polygonatum* species.

| Region | Number of nucleotide Sites | Total number of sites (excluding sites with gaps) | Indel diversity | Total number of Indel sites | Total number of indels events | Nucleotide diversity |
|---|---|---|---|---|---|---|
| *rps16-trnQ-UUG* | 1330 | 492 | 1.131 | 64 | 5 | 0.01937 |
| *trnS-GCU-trnG-UCC* | 1512 | 1062 | 7.076 | 333 | 27 | 0.01012 |
| *trnT-UGU-trnL-UAA* | 907 | 596 | 3.158 | 115 | 15 | 0.00850 |
| *trnV-GAC-rps7* | 2716 | 2642 | 1.789 | 74 | 8 | 0.00059 |
| *ndhC-trnV-UAC* | 1677 | 1606 | 3.146 | 71 | 8 | 0.00749 |
| *accD-psaI* | 257 | 241 | 0.105 | 2 | 1 | 0.00859 |
| *matK-rps16* | 1482 | 1334 | 5.181 | 141 | 20 | 0.00881 |
| *rpl32-trnL-UAG* | 961 | 892 | 1.661 | 57 | 6 | 0.01766 |
| *trnC-GCA-petN* | 886 | 872 | 2.491 | 14 | 9 | 0.01272 |
| *petA-psbJ* | 1111 | 1104 | 2.819 | 97 | 12 | 0.00930 |
| *ccsA* | 972 | 972 | 0.000 | 0 | 0 | 0.00502 |
| *ycf1* | 5745 | 866 | 0.105 | 24 | 1 | 0.00047 |

Table S10 Mean node ages and their 95% HPD intervals (millions of years ago, Ma) for nodes.

| Node | Mean age (Ma) | 95% HPD |
|---|---|---|
| 1 | 8.76 | 4.10–10.24 |
| 2 | 11.80 | 8.27–15.78 |
| 3 | 11.52 | 7.54–15.45 |
| 4 | 14.71 | 11.32–18.57 |
| 5 | 16.56 | 13.57–20.56 |
| 6 | 41.68 | 30.97–57.29 |
| 7 | 57.80 | 57.37–63.85 |
