# Supplementary material for: Comparative Analysis of Chloroplast Genome and New Insights Into Phylogenetic Relationships of Polygonatum and Tribe Polygonateae
Source: Front Plant Sci. 2022 Jun 24;13:882189. doi: 10.3389/fpls.2022.882189 (PMC9263837; doi:10.3389/fpls.2022.882189)
Supplement: Supplementary file 3 [file Image_1.PDF]

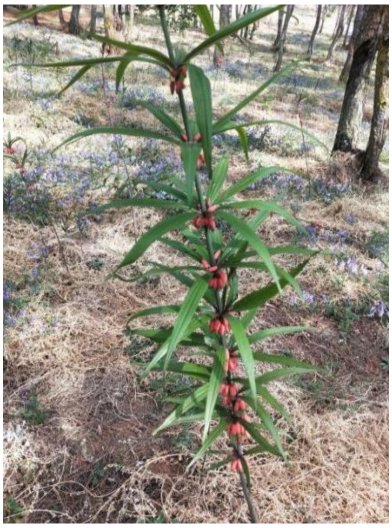

P. kingianum

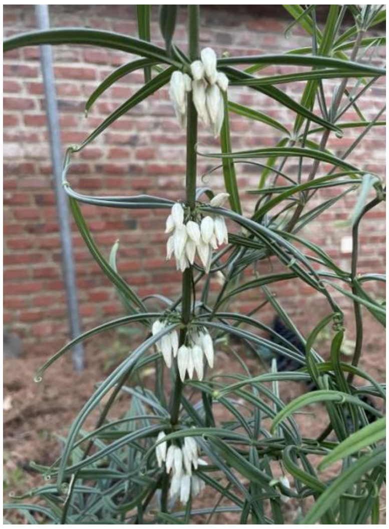

P. cirrhifolium

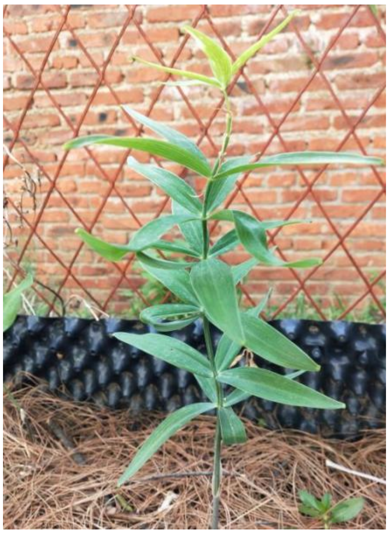

P. sibiricum 1

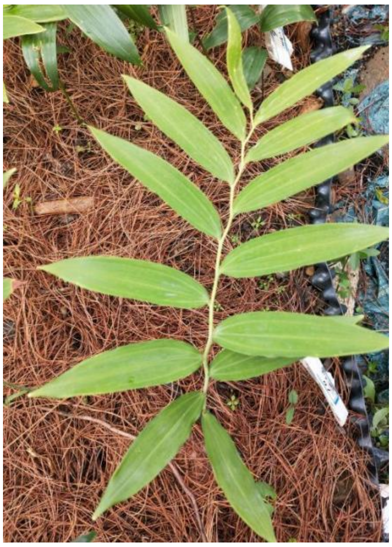

P. cyrtonema

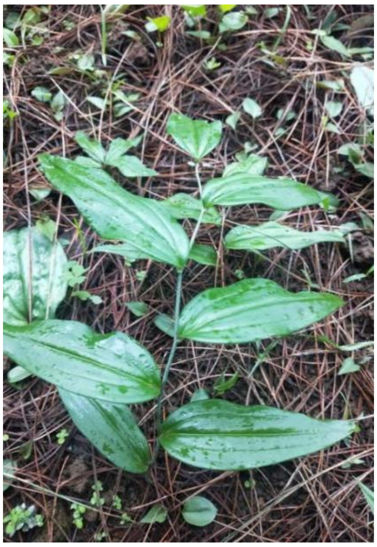

P. alternicirrhosum

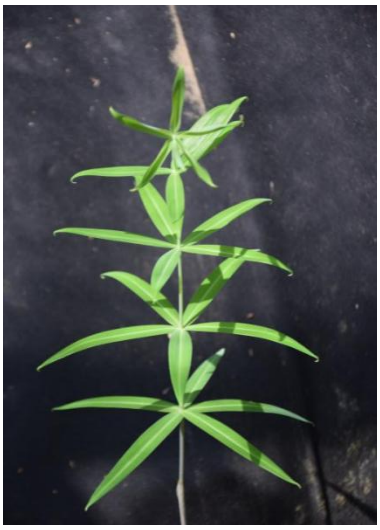

P. sibiricum 2

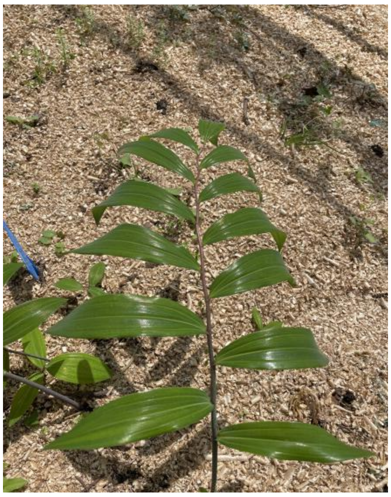

P. filipes

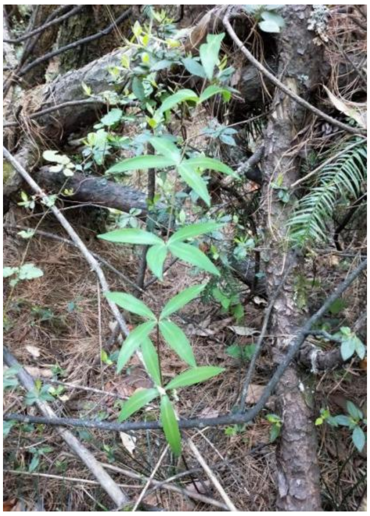

P. uncinatum

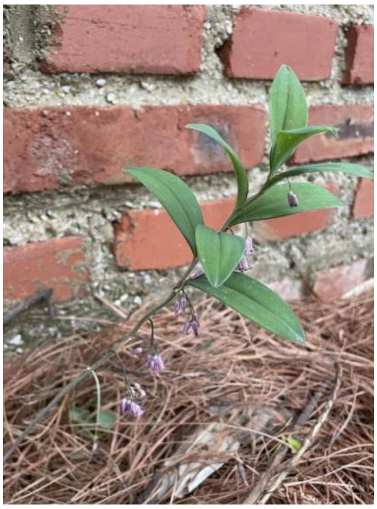

P. prattii

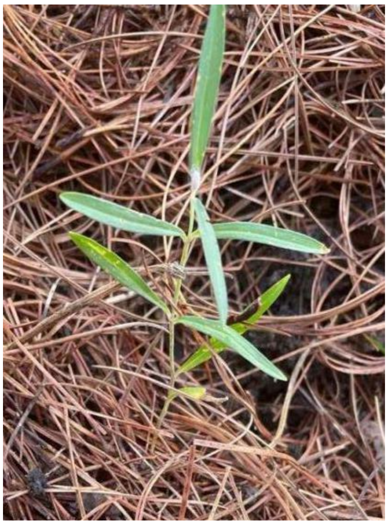

P. hookeri

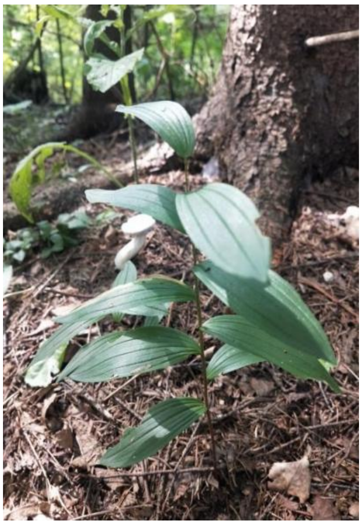

P. humile

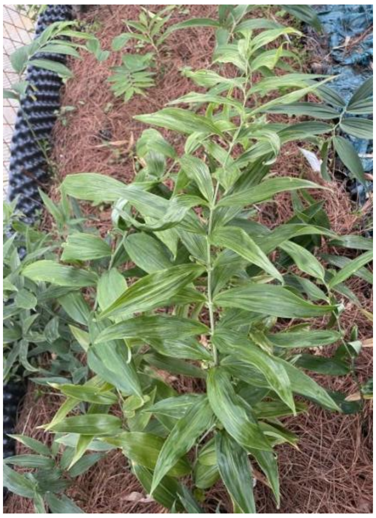

P. hunanense

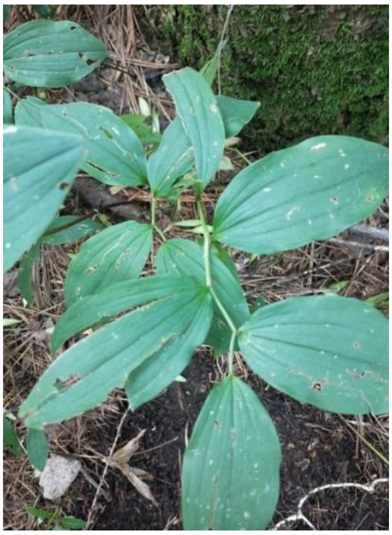

P. involucratum

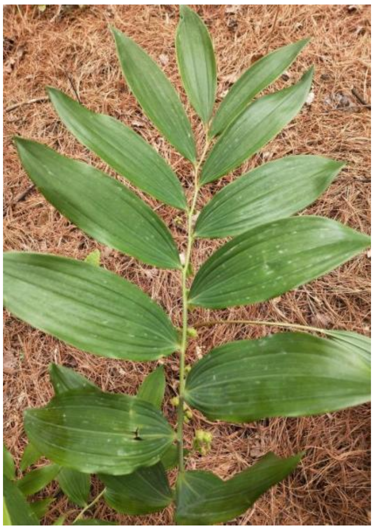

P. odoratum

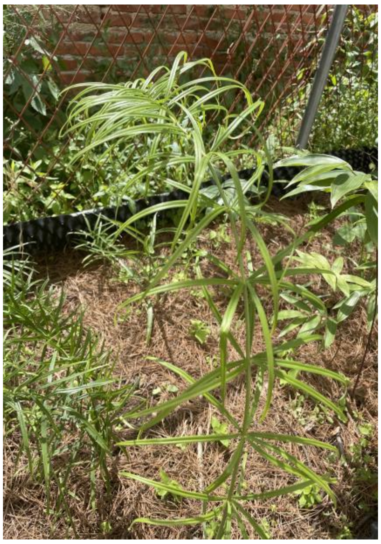

P. zanlanscianense

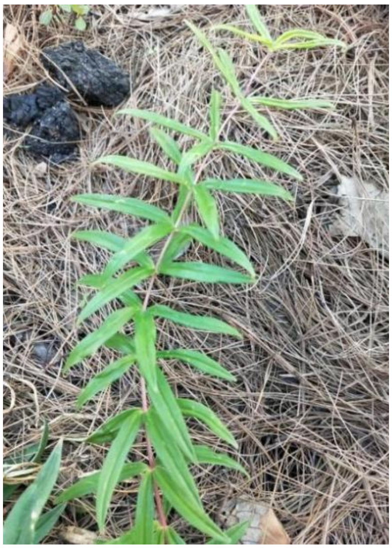

P. stewartianum

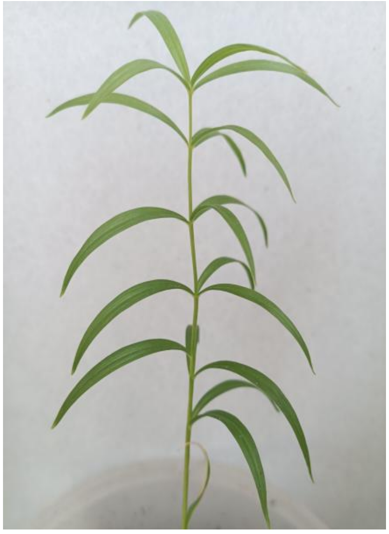

P. stenophyllum

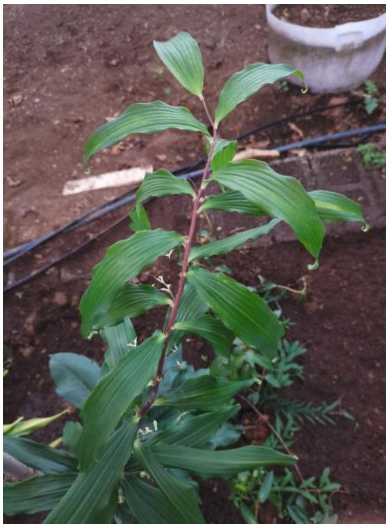

P. franchetii

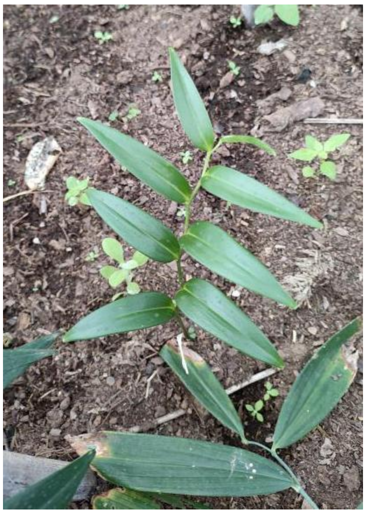

P. mengtzense

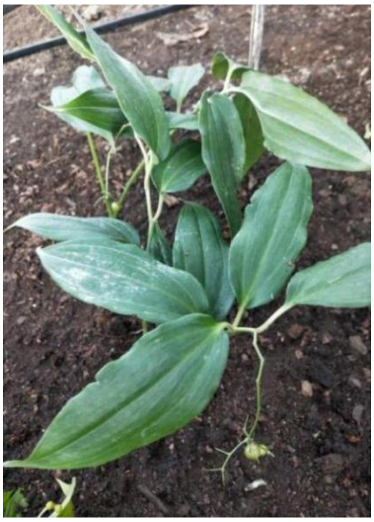

Maianthemum fuscum

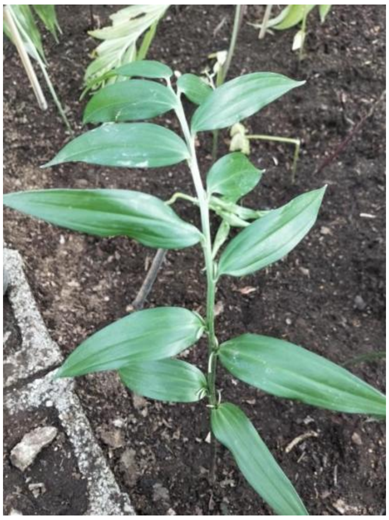

Disporopsis pernyi

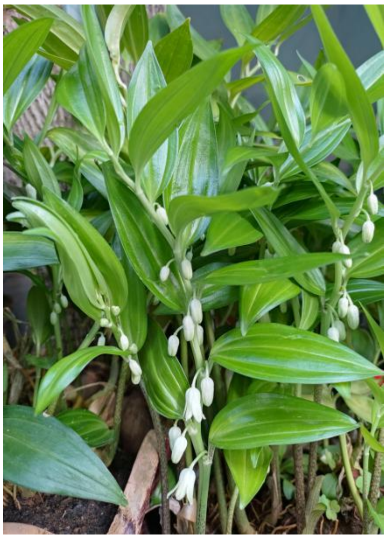

Disporopsis aspersa

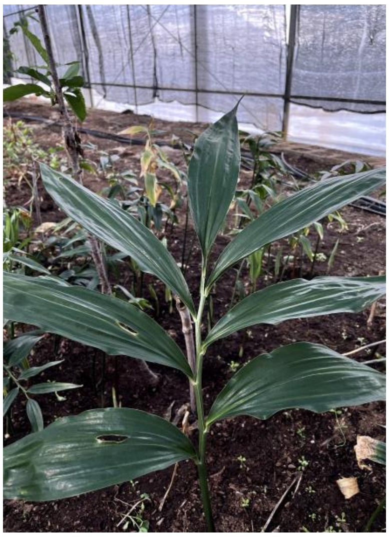

Disporopsis longifolia

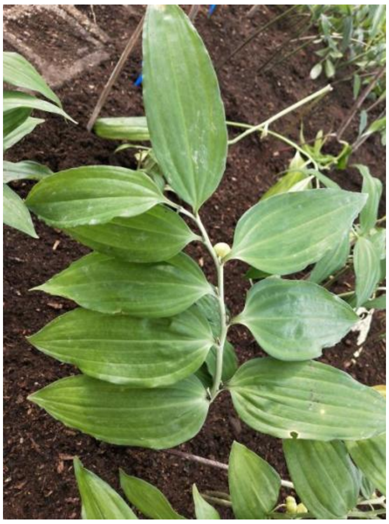

Disporopsis fuscopicta

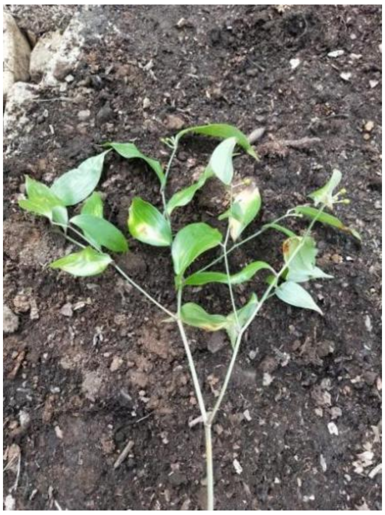

Disporum megalanthum

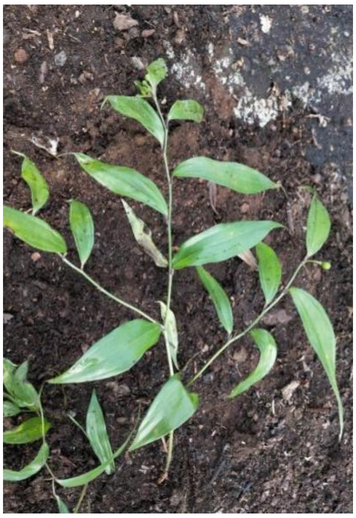

Disporum cantoniense

Fig. S1 Pictures of some plants.

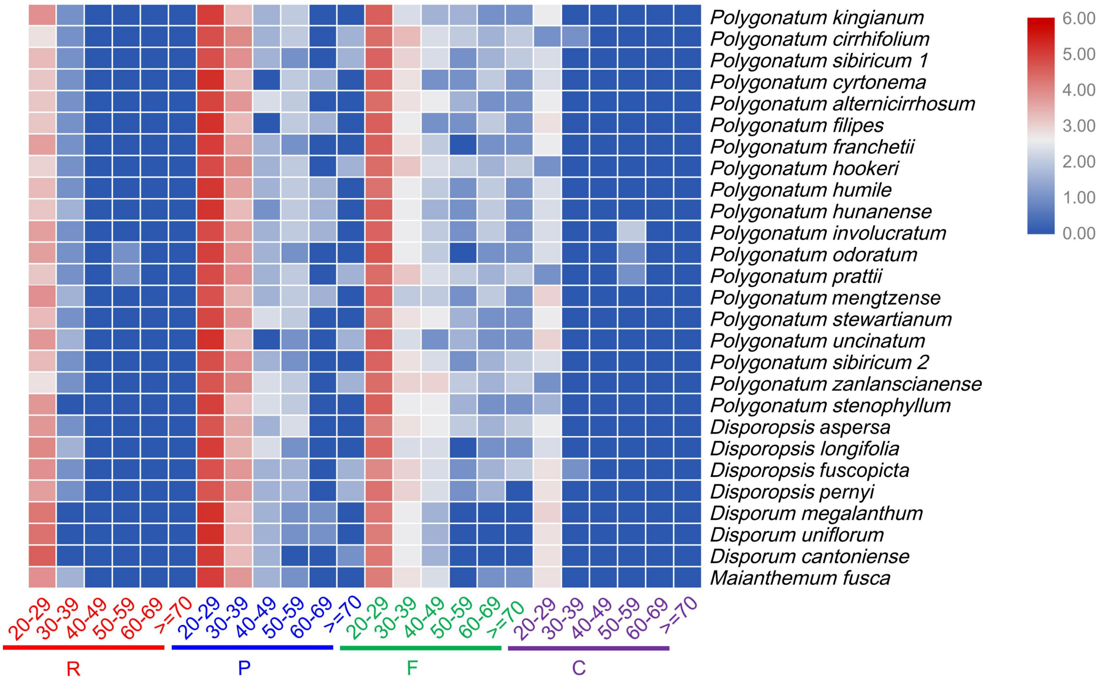

Fig. S2 Repeat sequences detected in the cp genome. The heat-map of four types of tandem repeated sequences in the 27 cp genomes. P, F, C and R indicate the repeat types: R (Reverse repeats), P (Palindromic repeats), F (Forward repeats), C (Complement repeats).

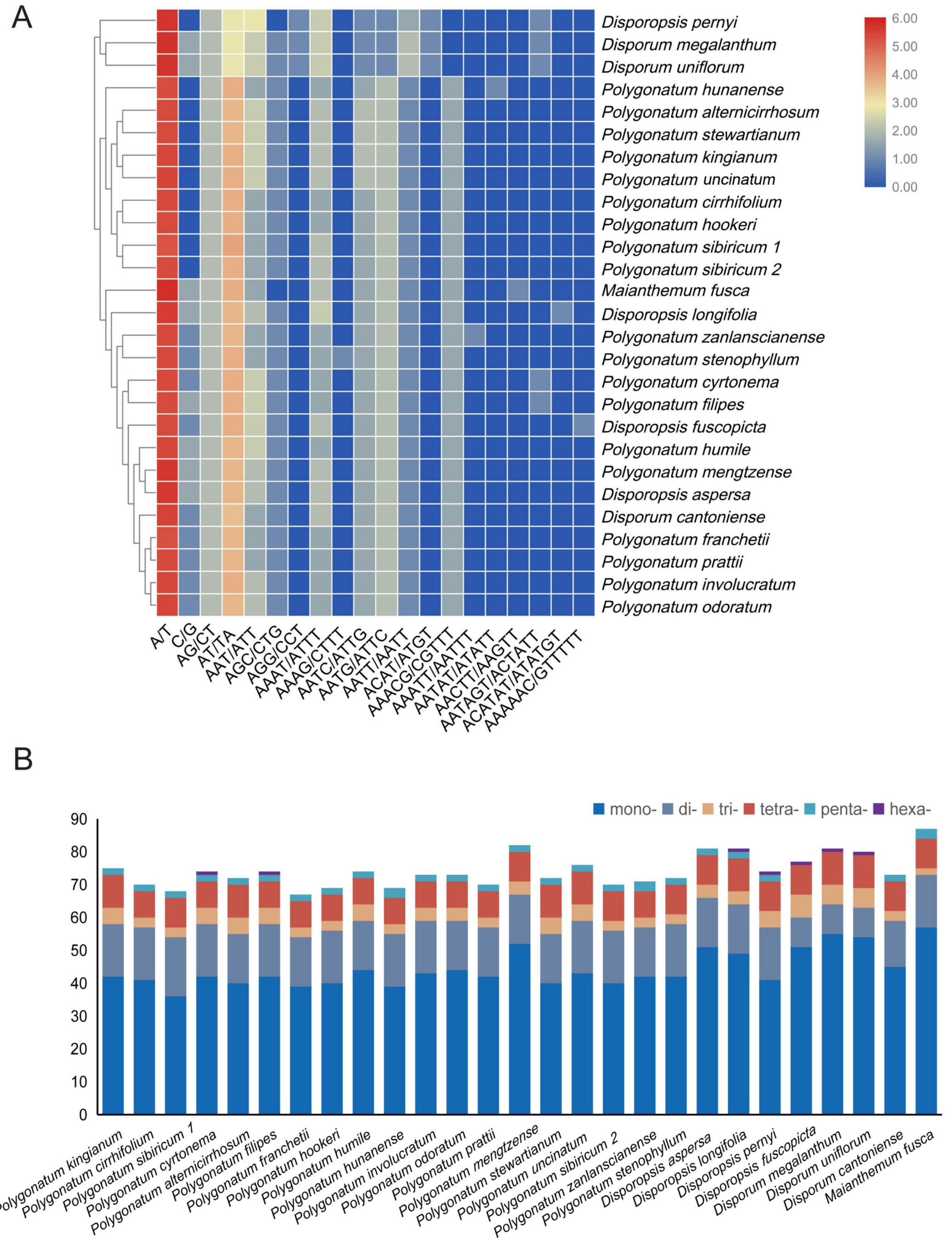

Fig. S3 The number and type of SSRs in the 27 cp genomes. (A) For each species, the heat- map of SSRs in the 27 cp genomes. (B) Number of types of SSRs presented in 27 cp genomes.

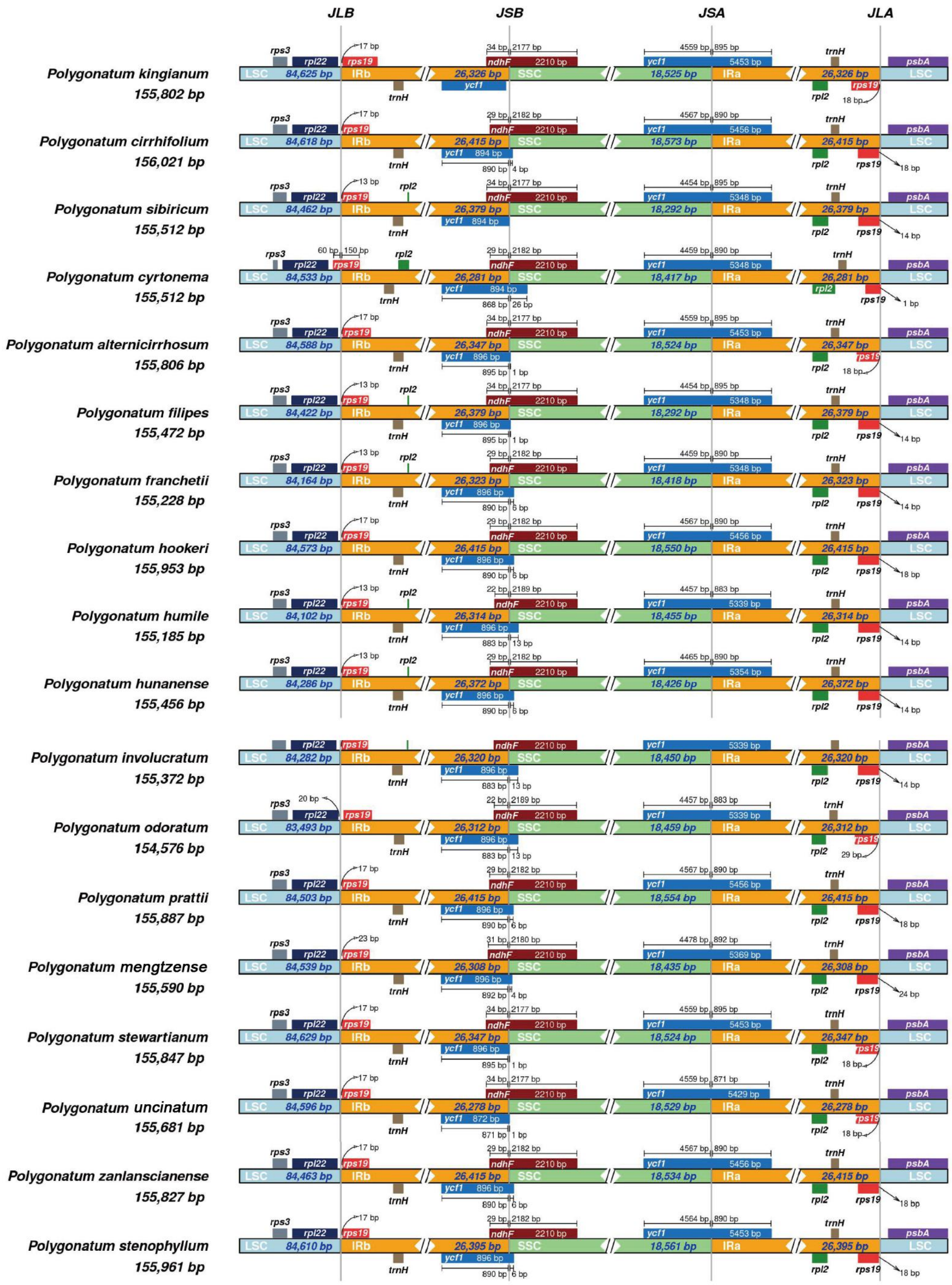

Fig. S4 Comparisons of the borders of LSC, SSC, and IRa/b regions among the 18 *Polygonatum* plastid genomes. The numbers above represent the distance between the gene ends and the borders sites, and the numbers below represent the length of the LSC, SSC, and IRa/b regions. This Figure is not to scale.

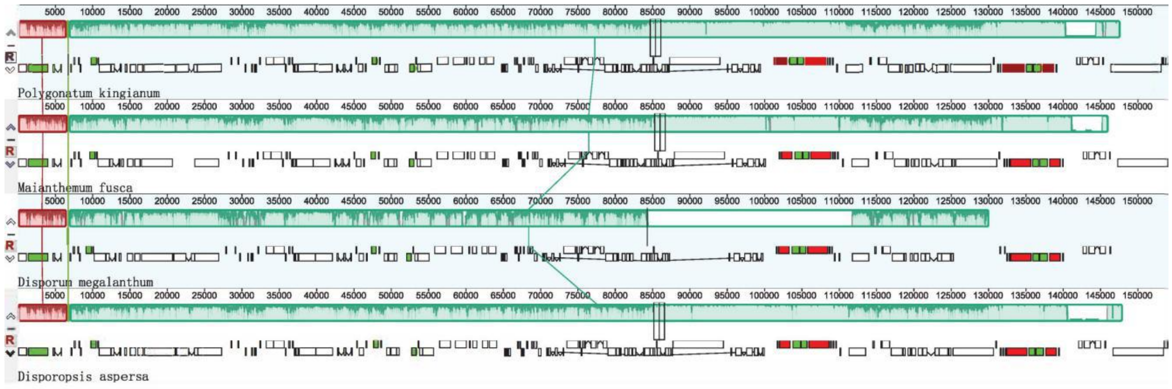

Fig. S5 The cp genome alignment analysis of *Polygonatum*, *Disporopsis, Maianthemum*, and *Disporum.*

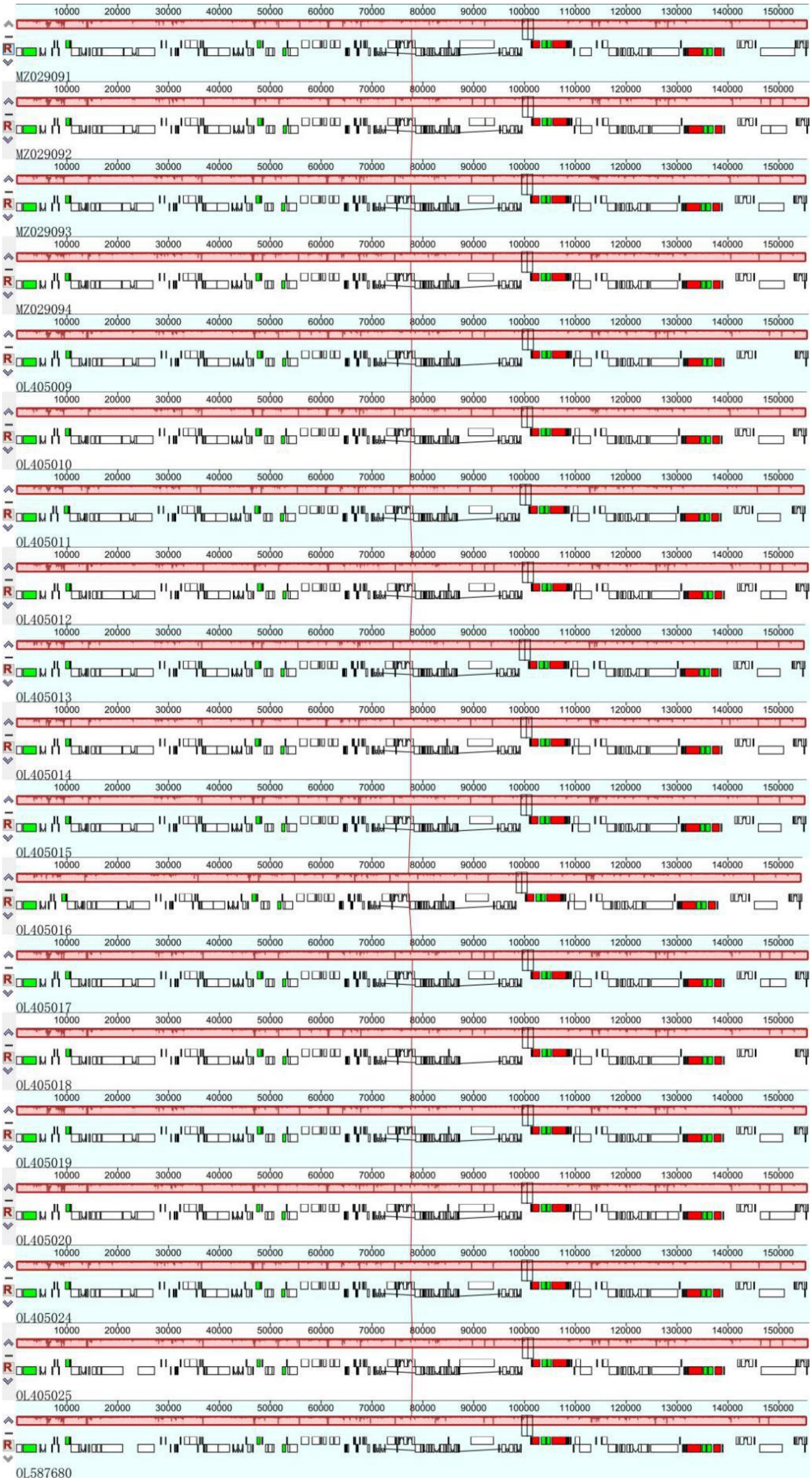

Fig. S6 The complete genome alignment analysis of *Polygonatum* species.

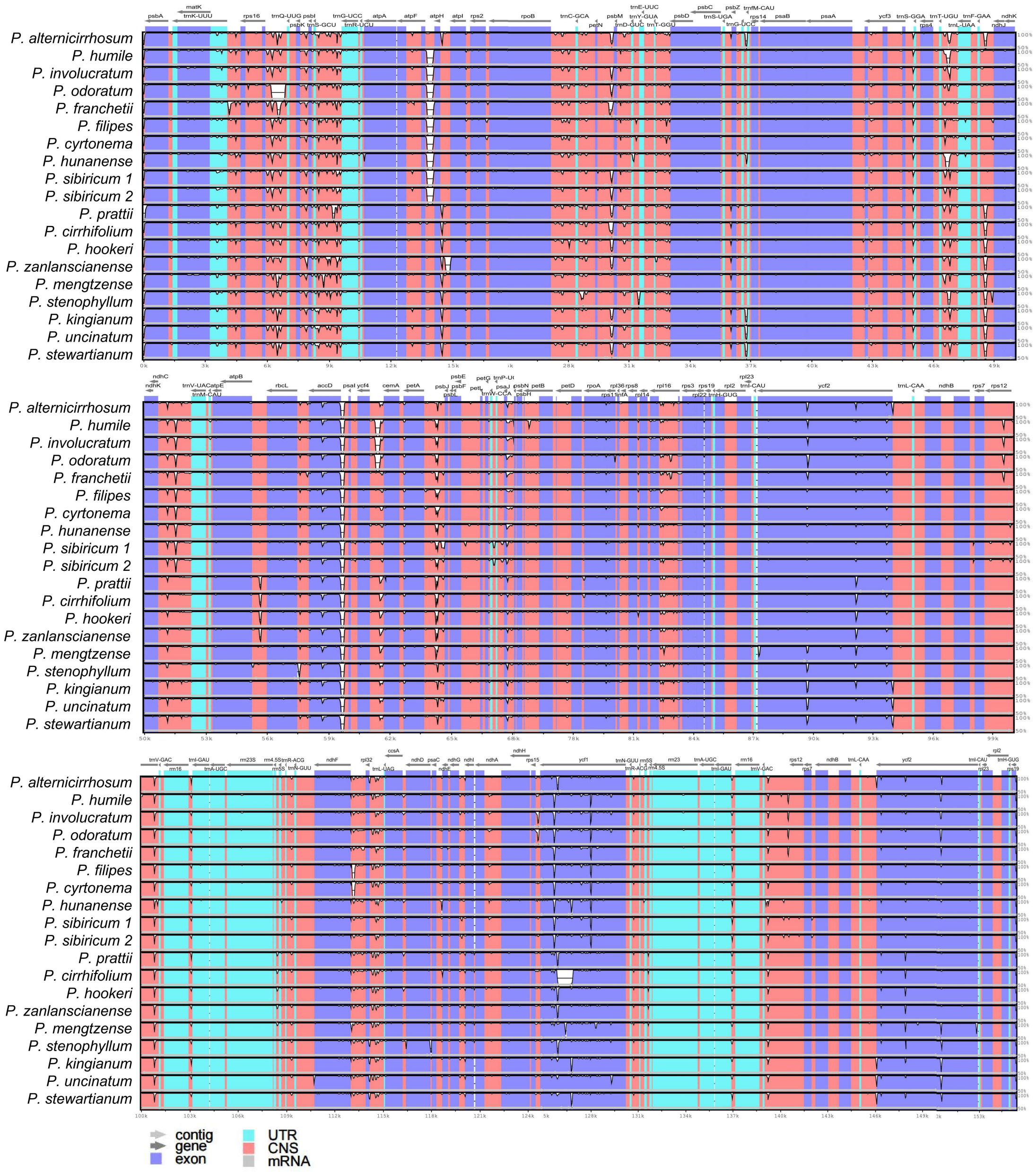

Fig. S7 Global comparison of complete genomes of *Polygonatum*. Coding and non-coding regions are colored in blue and red, respectively.

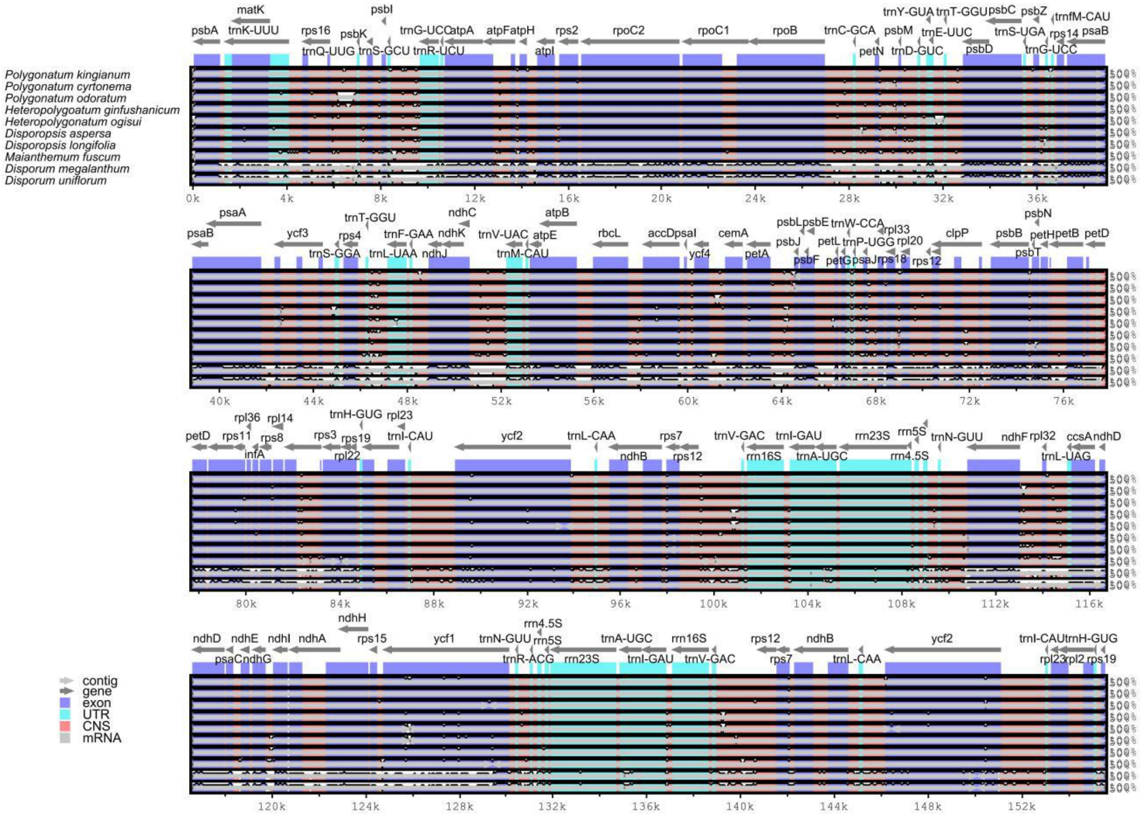

Fig. S8 Global comparision of complete genomes of *Polygonatum*, *Heteropolygonatum, Disporopsis, Maianthemum*, and *Disporum.*

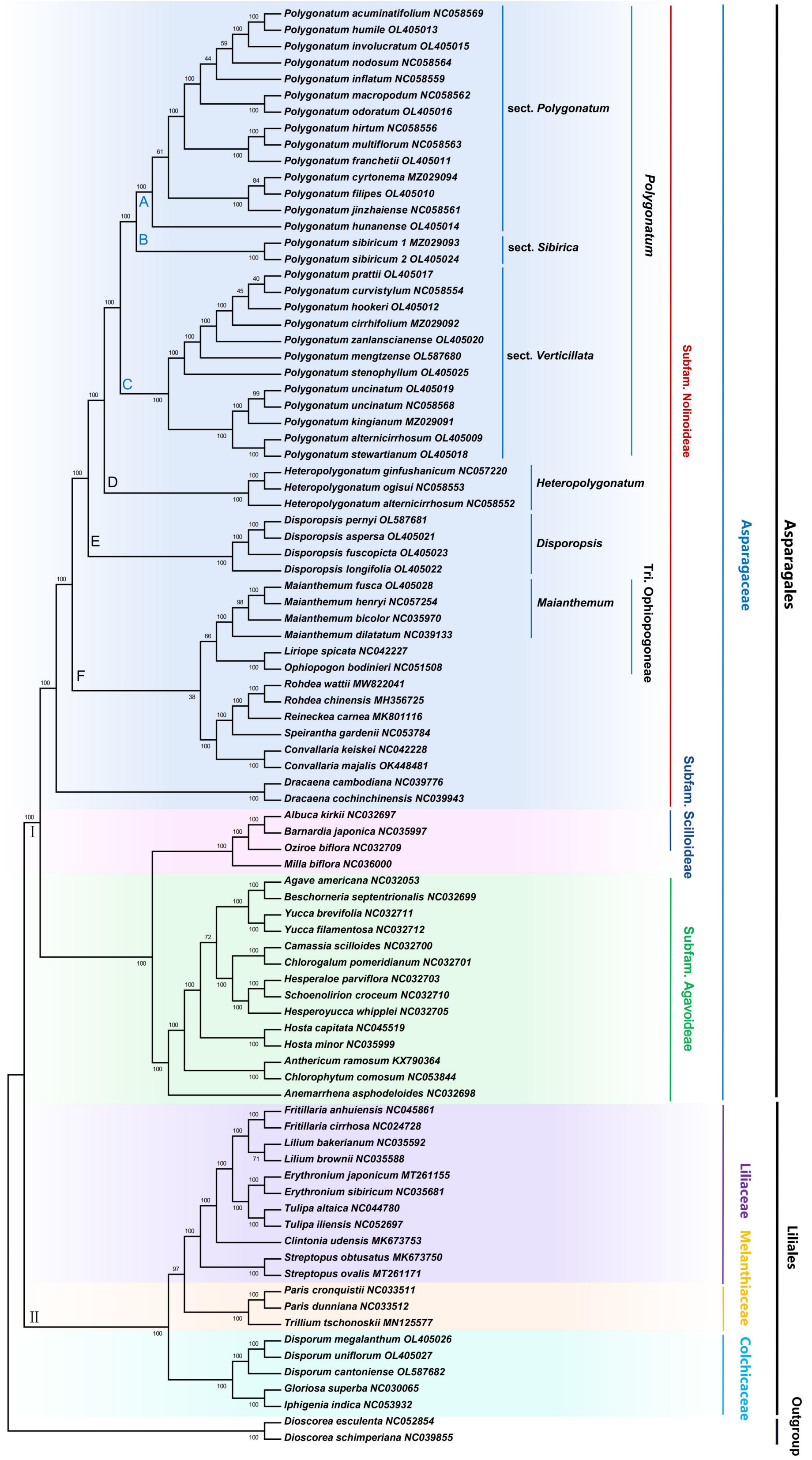

Fig. S9 Neighbor-Joining phylogenetic tree based on complete cp genome. *Dioscorea esculenta* and *D. schimperiana* were selected as outgroups. Numbers at nodes are bootstrap support values.
